# A novel *Staphylococcus aureus cis–trans* type I toxin–antitoxin module with dual effects on bacteria and host cells

**DOI:** 10.1093/nar/gky1257

**Published:** 2018-12-14

**Authors:** Noëlla Germain-Amiot, Yoann Augagneur, Emilie Camberlein, Irène Nicolas, Valérie Lecureur, Astrid Rouillon, Brice Felden

**Affiliations:** 1 Université de Rennes 1, Inserm, BRM (Bacterial Regulatory RNAs and Medicine) UMR_S 1230, 35000 Rennes, France; 2 Université de Rennes 1, CHU Rennes, Inserm, EHESP, Irset (Institut de recherche en santé, environnement et travail) UMR_S 1085, 35000 Rennes, France

## Abstract

Bacterial type I toxin–antitoxin (TA) systems are widespread, and consist of a stable toxic peptide whose expression is monitored by a labile RNA antitoxin. We characterized *Staphylococcus aureus* SprA2/SprA2_AS_ module, which shares nucleotide similarities with the SprA1/SprA1_AS_ TA system. We demonstrated that SprA2/SprA2_AS_ encodes a functional type I TA system, with the *cis*-encoded SprA2_AS_ antitoxin acting in *trans* to prevent ribosomal loading onto SprA2 RNA. We proved that both TA systems are distinct, with no cross-regulation between the antitoxins *in vitro* or *in vivo*. SprA2 expresses PepA2, a toxic peptide which internally triggers bacterial death. Conversely, although PepA2 does not affect bacteria when it is present in the extracellular medium, it is highly toxic to other host cells such as polymorphonuclear neutrophils and erythrocytes. Finally, we showed that SprA2_AS_ expression is lowered during osmotic shock and stringent response, which indicates that the system responds to specific triggers. Therefore, the SprA2/SprA2_AS_ module is not redundant with SprA1/SprA1_AS_, and its PepA2 peptide exhibits an original dual mode of action against bacteria and host cells. This suggests an altruistic behavior for *S. aureus* in which clones producing PepA2 *in vivo* shall die as they induce cytotoxicity, thereby promoting the success of the community.

## INTRODUCTION

*Staphylococcus aureus* is a serious human bacterial pathogen that causes life-threatening nosocomial and community-acquired infections (1). The success of a staphylococcal infection relies on the coordinated expression of a large set of genes involved in virulence, including toxins and surface proteins (2). Gene expression and the adjustment of bacterial physiology to external signals are both usually dependent on regulators such as regulatory RNAs (sRNAs, also called non-coding RNAs) or transcription factors (2,3). In *S. aureus*, sRNAs range in size from 50 to 500 nucleotides (nt), are usually non-coding, and control the expression of RNA targets that are involved in a wide range of biological processes which include virulence, antibiotic resistance, and central metabolism regulation (4–6). Recently, the sRNA repertoire was compiled into the Staphylococcal regulatory RNA database (SRD: http://srd.genouest.org/), which proposes a simple sRNA gene identifier (srn) for each molecule, and acts as a storehouse of useful information for the sRNA community (7). Most *S. aureus* sRNAs act by base-pairing with an RNA target to modulate translation initiation and/or RNA stability. They normally fall into two categories: *cis-* or *trans-*encoded sRNAs (4). *cis*-encoded sRNAs are transcribed from the opposite strand of another RNA (i.e. mRNA or sRNA), and they display perfect sequence complementarity with their target sequences. *trans*-encoded sRNAs are transcribed apart from their targets, and display only partial complementarity with them. Among the *cis*-encoded sRNAs, some studies evidenced the presence of small sense–antisense clusters, some of which can belong to toxin–antitoxin (TA) modules (7–13).

TA modules are divided into six types, and are widespread in bacteria and archaea (14,15). They share a common feature, which is that they all encode a stable toxin and a labile antitoxin which neutralizes its toxicity (15,16). To date, three types of TA systems (types I, II and III) have been identified in *S. aureus*, and their functions are slowly being elucidated (15,17–20). Among the *S. aureus* TA systems, two belong to type I TA modules and are expressed from pathogenicity islands, genomic islands acquired through horizontal gene transfer. These are SprA1/SprA1_AS_ and SprG1/SprF1 (17,18,20), referred to respectively in the SRD as Srn_3580_SprA1/Srn_3590_SprAs1 and Srn_3840_SprG1/Srn_3830_SprF1 (15). Type I TA modules are composed of a toxic peptide and an unstable antisense sRNA that inhibits toxin translation (21). These peptide toxins have multiple roles. These include small membrane damaging peptides inducing cell death, plasmid maintenance through post-segregational killing, global translation inhibition and commitment to persistence (15,19,21–24). Previous work in our lab revealed that in the Srn_3580_SprA1/Srn_3590_SprAs1 TA pair, Srn_3580_SprA1 expresses a single toxic peptide repressed, in *trans*, by a *cis*-encoded Srn_3590_SprA1_AS_ RNA antitoxin (18). The expression of Srn_3590_SprA1_AS_ sRNA prevents translation initiation of the toxin, allowing the bacterium to grow. More recently, the Srn_3830_SprF1/Srn_3840_SprG1 system was also characterized (20). This module produces two toxic peptides from the same Srn_3840_SprG1 transcript, each causing cell death. Toxicity is counterbalanced in *cis* by the Srn_3830_SprF1 antitoxin, which has dual functions because it represses toxicity at both the transcriptional and translational levels (16). Computational analysis revealed the presence of additional putative TA modules in *S. aureus* which share nucleotide and genetic organization similarities with either Srn_3580_SprA1/Srn_3590_SprAs1 or Srn_3840_SprG1/Srn_3830_SprF1 (13,17,20), and these have been reported in the SRD (7). Here, we describe the functional identification of a novel type I TA system Srn_4540_SprA2_AS_/Srn_4550_SprA2, which shares 75% sequence similarity with the SprA1/SprA1_AS_ TA system. We provide evidence that the Srn_4540_SprA2_AS_ antisense RNA binds Srn_4550_SprA2 at the ribosomal binding site through partial complementary base-pairing to prevent translation initiation. Based on mutational analysis and cell viability experiments, we demonstrate that SprA2 encodes PepA2, a cytotoxic peptide that also intracellularly triggers *S. aureus* death. We show here that there is no cross-regulation between the SprA2/SprA2_AS_ and SprA1/SprA1_AS_ TA modules, thus demonstrating their specificity. Finally, we also reveal that antitoxin expression is reduced during an osmotic stress and in a stringent medium. These events might be the signals that trigger PepA2 expression to influence colonization, spread and/or infection.

## MATERIALS AND METHODS

### Bacterial strains, growth, toxin induction, viability assays, and stress conditions

The bacterial strains and plasmids used in this study are listed in Table 1. Except where stated otherwise, cells were grown in brain heart infusion broth (BHI, Oxoid) or in tryptic soy broth (TSB, Oxoid) under agitation at 37°C. For all experiments, overnight cultures were diluted to an OD_600 nm_ of 0.1, and then monitored at different time points. For co-culture assays, species were mixed to a ratio of 50:50 to reach an OD_600nm_ of 0.05. When necessary, 5 μg/ml chloramphenicol and/or erythromycin was used, while kanamycin was used at 50 μg/ml. Toxin production was induced by the addition of either 100 nM or 1 μM anhydrotetracycline (aTc). Viability assays were conducted prior aTc induction and then 2.5 h afterwards. For these assays, cell density was adjusted to an OD_600nm_ of 0.02, 10-fold serial dilutions were performed with BHI, then 10 μl of each dilution was placed on BHI agar plates supplemented with the appropriate antibiotics. For the analysis of RNA levels while undergoing different stresses, *S. aureus* was cultured as previously described (9). Stresses were respectively induced by the addition of 10 mM H_2_O_2_ (for oxidative stress), HCl (to lower the pH to 5.5), or NaOH (to increase the pH to 9.5), or by changing the temperature to 18 or 42°C (cold and heat shocks). In addition, 60 ml of culture was centrifuged at 4500 rpm for 8 min at room temperature, and the pellets resuspended in TSB supplemented with 1 M NaCl (osmotic stress); in NZM medium (stringent response); or in fresh TSB as a control.

**Table 1.** Bacterial strains and plasmids used in this study

Strains and plasmids	Characteristics	References
***S. aureus* strains**		
Newman	Methicillin-sensitive *S. aureus* strain	(58)
*ΔsprA2ΔsprA2_AS_*	Newman strain deleted for *sprA2* and *sprA2_AS_* and containing *Km^R^* cassette	This study
N315	Erythromycin-resistant *S. aureus* strain	(59)
**Other strains**		
XL-1 Blue	*recA1 endA1 gyrA96 thi-1 hsdR17 supE44 relA1 lac*[F’proAB *lacI^q^ ZΔM15*Tn*10* (Tet^r^)]	Stratagene
EC101	Kanamycin-resistant *Escherichia coli* strain	(60)
*Salmonella typhimurium*	*Salmonella enterica* serovar Typhimurium LT2	(61)
*E. faecium* Aus004	*Enterococccus faecium* strain Aus004	(62)
**Plasmids**		
pALC	pALC2073 with the tetracycline-inducible *Pxyl/tetO* promoter with *Amp^R^* in *E. coli* and *Chlo^R^*	(63)
pALC_*sprA1*	pALC2073 with *sprA1* cloned under *Pxyl/tetO* promoter	This study
pALC_*sprA2*	pALC2073 with *sprA2* cloned under *Pxyl/tetO* promoter	This study
pALC_*sprA2^A53T,T54A^*	pALC2073 with *sprA2^A53T,T54A^* cloned under *Pxyl/tetO* promoter	This study
pALC_*sprA2^G52C^*	pALC2073 with *sprA2^G52C^* cloned under *Pxyl/tetO* promoter	This study
pCN35c	Modified high-copy-number shuttle with *Amp^R^* in *E. coli* and *Chlo^R^* instead of *Erm^R^* in *S. aureus*	(29)
pCN35c_*sprA2*Flag	pCN35c with *sprA2* under its own promoter and fused with *flag* at the 5′ end	This study
pCN35c_*sprA2*Flag*sprA2_AS_*	pCN35c_*sprA2*Flag containing *sprA2_AS_* under its own promoter	This study
pCN35c_*sprA1_AS_*	pCN35c with *sprA1_AS_* under its own promoter	This study
pCN35c_*sprA2_AS_*	pCN35c with *sprA2_AS_* under its own promoter	This study
pCN41	Vector carrying *blaZ*, reporter gene and conferring *Amp^R^* in *E. coli* and *Erm^R^* in *S. aureus*	(29)
pCN41_P*sprA2_AS_*	pCN41c with *blaZ* under the control of P*sprA2_AS_*	This study
pCN35	High-copy-number shuttle with *Amp^R^* in *E. coli* and *Erm^R^* in *S. aureus*	(29)
pCN35___*sprA1_AS_*	pCN35 with *sprA1_AS_* under its own promoter	This study
pCN35___*sprA2_AS_*	pCN35 with *sprA2_AS_* under its own promoter	This study

### Total RNA extractions

Cells were harvested by centrifugation at 4500 rpm for 10 min at 4°C, and pellets washed with 500 μl lysis buffer (20 mM sodium acetate, 1 mM EDTA, 0.5% SDS, pH 5.5). Cell pellets were broken using acid-washed glass beads (Sigma) in the presence of phenol (pH 4) in a FastPrep FP120 cell disrupter (MP Biomedicals) for 30 s at a power setting of 6.5. Lysates were centrifuged at 16 000 g for 10 min at 4°C. Total RNAs were extracted with phenol/chloroform and precipitated overnight at −20°C with ethanol supplemented with 0.3 M sodium acetate.

### Northern blot analysis, RNA stability assays and RACE mapping

Northern blots were performed as previously described (9). Briefly, 10 μg of total RNAs were loaded and separated in 8% polyacrylamide/8M urea gels. The RNAs were probed with ^γ32^P 5′-end labeled oligonucleotides (Supplementary Table S1) and detected using a Typhoon FLA 9500 scanner (GE Healthcare). RNA half-life determinations were done using 200 μg/ml rifampicin, with or without 100 nM aTc. *Staphylococcus aureus* Newman strain with pALC_*sprA2* + pCN35_*sprA2_AS_* was cultured for 2 h, and SprA2 induced by the addition of aTc. After 30 min induction, rifampicin was added to the culture and total RNAs extracted at different time intervals. RNA amounts were quantified using a Typhoon FLA 9500 scanner (GE Healthcare) and tmRNA as an internal loading control. RACE mapping (rapid amplification of cDNA ends) was performed as previously described (20) using the primers listed in Supplementary Table S1.

### Western blots

Recombinant plasmids (Table 1) were transformed in *S. aureus* as previously described (25). *S. aureus* was cultured in BHI broth supplemented with the appropriate antibiotics at 37°C and under agitation until reaching an appropriate OD_600nm_ (see Results). Cells were harvested by centrifugation, and cellular protein extracts were prepared with protease inhibitors as previously described (9). Proteins from the supernatants were precipitated with trichloroacetic acid (TCA), then washed with acetone as previously described (9). Samples were separated on 16% Tricine–SDS-PAGE gels and transferred onto Hybond P PVDF membranes (Amersham). After overnight blocking at 4°C under gentle agitation, the membranes were incubated with horseradish peroxidase-conjugated (HRP) anti-FLAG antibodies (Sigma). The membranes were revealed using an ECL Prime western blotting detection kit (Amersham) and scanned with an ImageQuant LAS 4000 (GE).

### Peptide synthesis, hemolytic and cytotoxic assays

PepA2 and PepA1 peptides were synthesized by automated solid-phase synthesis with the kind help of Dr Baudy-Floc’h's COrInt group (ISCR-UMR 6226, University of Rennes 1), and characterized as described previously (26). High-performance liquid chromatography (HPLC) analysis was done to ensure that the purity of the final products was above 95%, and their expected molar mass was confirmed by matrix-assisted laser desorption/ionization time-of-flight mass spectrometry (MALDI-TOF).

Cytotoxic assays were conducted using human blood provided by the Etablissement Français du Sang from at least three independent samples (EFS Rennes). For hemolytic assays, blood was washed and then resuspended at 5% (v/v) in phosphate-buffered saline (PBS). The peptide solutions were added at 2× concentrations to V-bottom 96-well plates after which 75 μl of 5% blood was added. The negative control was performed using PBS instead of peptides, while 100% hemolysis was assessed using 1% Triton X-100. Plates were incubated for 2 h at 37°C, then centrifuged at 1400 rpm for 15 min at room temperature. Finally, 100 μl supernatant was transferred into a flat-bottom 96-well plate and the OD_414 nm_ measured to assess the presence of released haemoglobin.

Polymorphonuclear neutrophils (PMNs) were isolated using the EasySep™ Direct Human Neutrophil Isolation kit (Stemcell) per the manufacturer's recommendations. Neutrophils were resuspended in RPMI medium supplemented with 2% fetal veal serum (FVS), then 20 000 neutrophils were distributed in each of the 96 wells of a tissue culture plate. Cells were incubated for 2 h at 37°C and 5% CO_2_ with increasing concentrations of PepA2. Neutrophils were harvested by centrifugation at 350 g for 8 min at 4°C, washed with RPMI 2% FVS, then stained with 50 μg/ml propidium iodide (PI) (Sigma-Aldrich) for 15 min in the dark at room temperature. After washing, cell viability was determined and analysed on a BD Biosciences LSR II cytometer using FACSDiva™ software. Since PI is not permeant to live cells, the percentage of unstained PMNs corresponds to the viable cell count.

### *In vitro* transcription and translation assays

*In vitro* transcription was conducted from PCR-amplified fragments cloned into pUC19 under the control of the T7 promoter, and containing a HaeIII restriction site at the sequence's 3′ end. Plasmids were linearized with HaeIII and subjected to *in vitro* transcription using the MEGAscript T7 kit (Thermo Fisher) according to the manufacturer's recommendations. RNAs were gel-purified, eluted, and precipitated with ethanol in the presence of 0.3 M sodium acetate. The *in vitro* translation assay was performed with ^[35S]^-methionine using an *E. coli* S30 extract system for linear templates (Promega) as per the manufacturer's instructions.

### Electrophoretic mobility shift assays and toeprints

RNAs were probed with oligonucleotides labelled at the 5′ ends with ^γ32^P. For electrophoretic mobility shift assays, RNAs were denatured in buffer (80 mM HEPES pH 7.5, 330 mM KCl, 4 mM MgCl_2_) for 2 min at 80°C, chilled on ice, then refolded for 20 min at 20°C. Binding was conducted in 10 μl for 30 min at 30°C. 0.1 pmol of labelled RNA was incubated with increasing amounts of unlabeled RNA. The specificity of the complex was assessed by adding an excess of unlabeled RNA (for concentrations used, see Figures 6 and 8). Samples were supplemented with glycerol to a final concentration of 10%, then loaded onto native 8% polyacrylamide gels containing 5% glycerol. Electrophoresis was performed in 0.5× Tris–borate–EDTA buffer at 4°C and 100 V. The results were analysed on a PhosphorImager and *K*_d_ values determined accordingly.

For toeprinting assays, annealing mixtures containing 0.25 pmol unlabelled SprA2 and 1.5 pmol labelled primer in a buffer (20 mM Tris-acetate pH 7.5, 60 mM NH_4_Cl, 1 mM DTT) were incubated for 1 min at 90°C, and then quickly chilled on ice for 1 min. Renaturation was done in 10 mM MgCl_2_ at 20°C for 15 min. The influence of SprA2_AS_ was assayed by adding 0.25 pmol to the annealing mixtures, then incubating for 5 min at 37°C. Purified *Escherichia coli* 70S ribosomes were diluted in the reaction buffer in the presence of 1 mM MgCl_2_ then activated for 15 min at 37°C. For each sample, 4 pmol purified 70S ribosomes were added, followed by 5 min incubation at 37°C and adjustment of the MgCl_2_ concentration to 10 mM. After 10 min at 37°C, 10 pmol tRNA^fMet^ was added and the samples were incubated for 5 min at 37°C. The cDNAs were synthesized using 4 units AMV RT (New England Biolabs) for 15 min at 43°C. The reaction was stopped by adding 10 μl loading buffer II (Ambion). The samples were incubated for 5 min at 95°C, then placed on ice. The cDNAs were loaded and separated onto 8% urea–PAGE gels. The toeprint position on SprA2 was determined by DNA sequencing, and the results were analysed on a PhosphorImager.

### Reporter gene experiments

Overnight cultures were adjusted to an OD_600 nm_ of 0.1. For each time point, cells were centrifuged and resuspended in 1× PBS to obtain an equal cell density throughout the assay. Cell lysis was performed using 0.7 mg/ml lysostaphin, 0.2 U/μl benzonase and 0.1 mM MgCl_2_ at 37°C for 20 min. After adding 0.25 mg/ml nitrocefin (a ß-lactamase substrate), ß-lactamase activity was quantified on a BioTek instrument every 10 min for 40 min at 492 nm. This activity was normalized against the protein quantities determined by a Bradford assay.

## RESULTS

### Both *sprA2* and *sprA2_AS_* genes are expressed in *Staphylococcus aureus*

**Figure 1.**
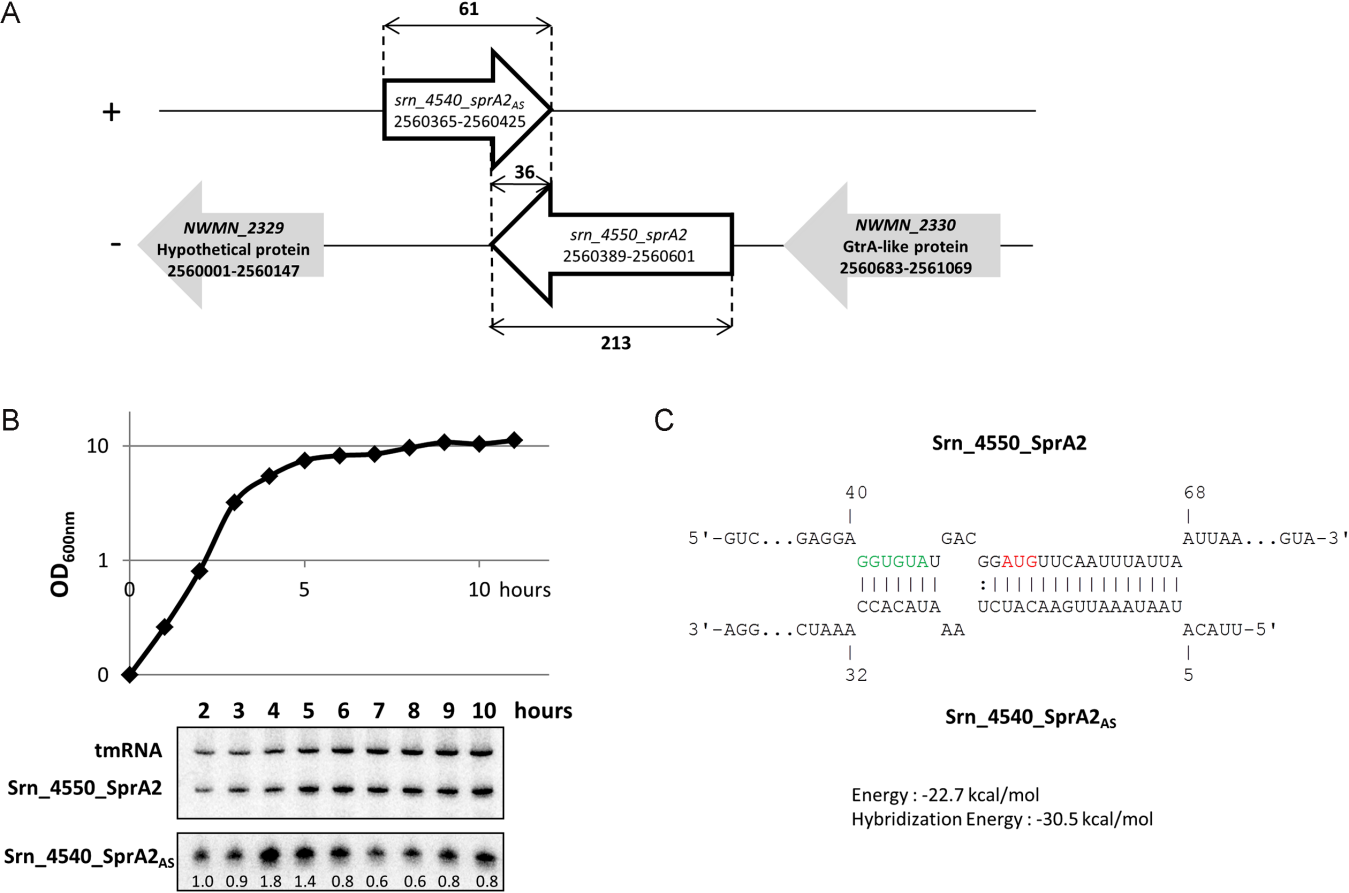
The putative type I toxin–antitoxin module Srn_4550_sprA2/Srn_4540_sprA2_AS_ is expressed in *Staphylococcus aureus* Newman. (**A**) Schematic of the genetic organization of the *srn_4550_sprA2/srn_4540_sprA2*_*AS*_locus. (**B**) Srn_4550_sprA2/Srn_4540_sprA2_AS_ expression profile during growth in BHI. Growth was monitored by measuring the optical density at 600 nm (OD_600nm_) every hour (upper chart). Expression was monitored by northern blotting, with tmRNA as an internal loading control (lower chart), with the relative amount of Srn_4540_SprA2_AS_ RNA indicated under each band. (**C**) *In silico* best prediction of the molecular interaction between the two components made with intaRNA program. The putative ribosome-binding site is green and the start codon is red. The coordinates appearing on panels A and C were experimentally determined by RACE mapping.

The *S. aureus* Newman *srn_4540_sprA2_AS_/srn_4550_sprA2* locus was identified based on its 75% sequence similarity with the pair *srn_3580_sprA1/srn_3590_sprA1_AS_* (7,13,17) (Supplementary Figure S1). For simplicity, they are referred to here as SprA1, SprA2, SprA1_AS_ and SprA2_AS_. As shown in Figure 1A, the *sprA2/sprA2_AS_* pair is located in the core genome of *S. aureus* Newman, between genes encoding a hypothetical protein and one in the GtrA family. To demonstrate and study their expression during bacterial growth, we designed novel probes (Figure 1B). Growth conducted in BHI (upper chart) showed that both *srna* genes were transcribed and detected by northern blots. While the SprA2 levels were similar to those of the tmRNA control, the intensity of the SprA2_AS_ bands varied in a different manner than the control. We quantified each band to determine the relative SprA2_AS_ RNA levels. These increased by around 1.8-fold after 4 h growth, which corresponds to the post-exponential phase, which indicates that there were therefore only moderate SprA2_AS_ variations under these conditions. We then did RACE mapping to determine the 5′ and 3′ boundaries, confirming that both genes overlap in their 3′ regions (see Figure 1A for the coordinates resulting from RACE). We used IntaRNA software (27) for *in silico* exploration of the two RNA sequences. This showed that, as for the *sprA1/sprA1_AS_* pair, these two RNAs could interact in *trans* with their 5′ regions through partial pairings (Figure 1C), with SprA2_AS_ binding SprA2 both at a putative ribosomal binding site and at its adjacent start codon, suggesting that it could indeed act as a type I TA system.

### SprA2/SprA2_AS_ is a functional type I toxin–antitoxin system that triggers *Staphylococcus aureus* cell death

**Figure 2.**
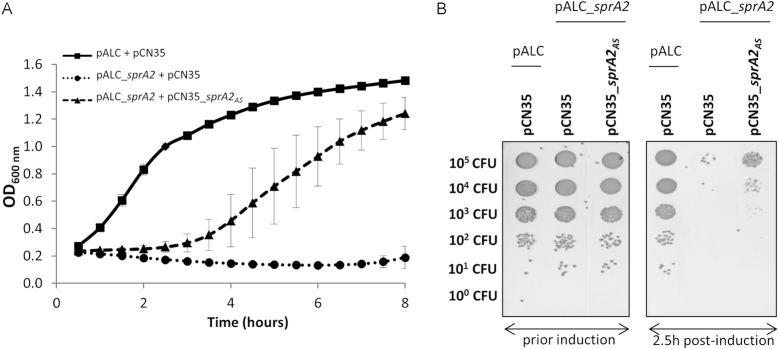
*sprA2/sprA2_AS_* encodes and expresses a functional toxin–antitoxin system. (**A**) Comparative growth curves after induction with 1 μM anhydrotetracycline (aTc) on *Staphylococcus aureus* Newman replicating empty plasmids pALC and pCN35 (squares); pALC_*sprA2* and pCN35 (circles); and pALC_s*prA2* and pCN35_*sprA2_AS_* (triangles). Growth was conducted in a Biotek instrument for 2.5 h, then expression was induced by aTc. Data points shown represent the growth after this addition, and are the mean of three independent biological replicates. (**B**) Assessment of cell viability by plating 10-fold serial dilutions, prior induction (left panel) or 2.5 h post-induction (right panel). All cultures (broth and agar) contain chloramphenicol and erythromycin concentrations appropriate for plasmid maintenance.

**Figure 3.**
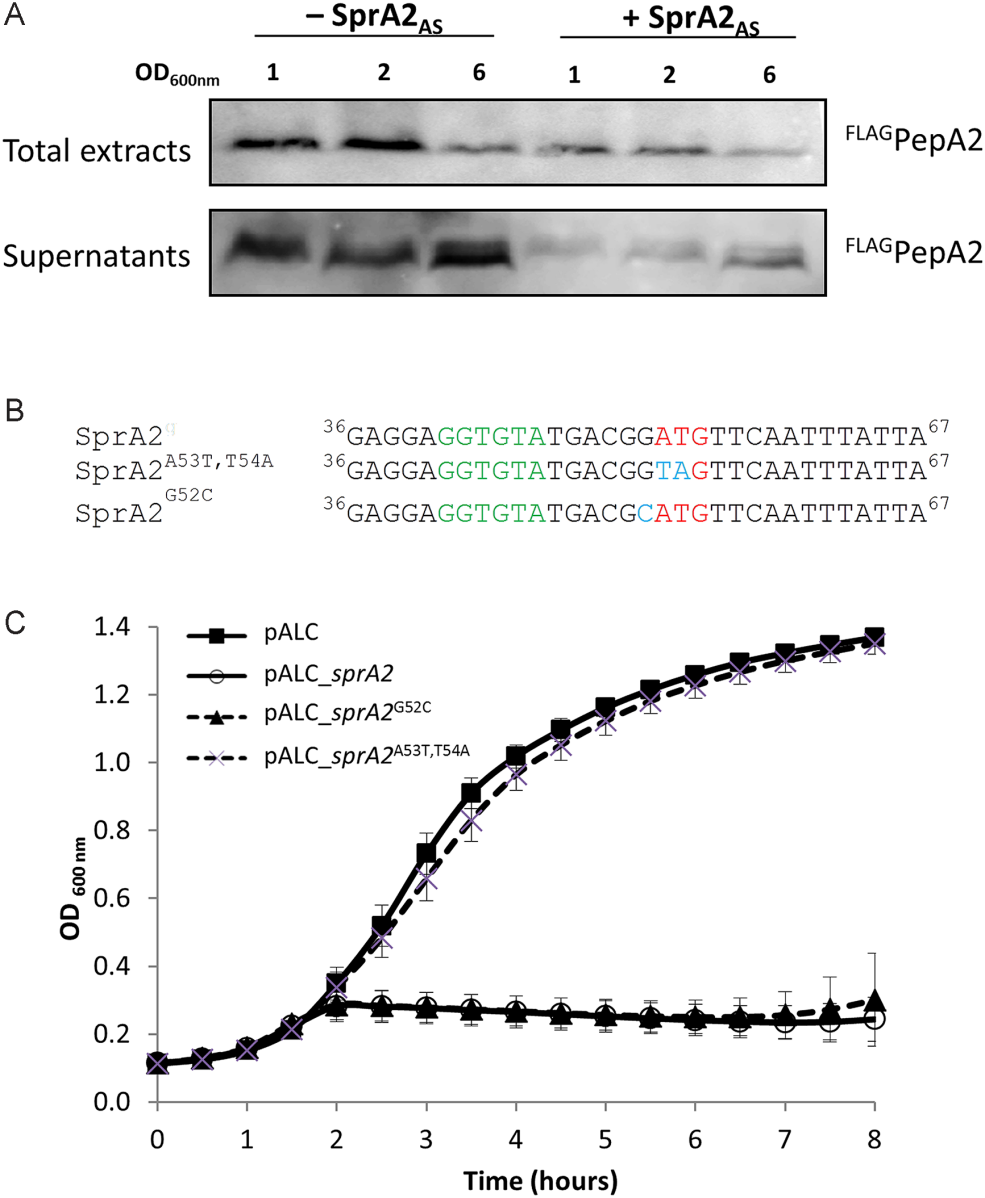
Bacterial toxicity is linked to the expression of PepA2. (**A**) *In vivo* expression of flagged PepA2, comparing *Staphylococcus aureus* Newman strains *ΔsprA2ΔsprA2*_*AS*_pCN35_*sprA2*Flag compared to Newman *ΔsprA2ΔsprA2_AS_* pCN35_*sprA2*Flag*sprA2_AS_*. (**B**) Point mutations were done in *sprA2* to avoid translation initiation or to modify RNA secondary structure. (**C**) Comparative growth curves of Newman strain containing. empty plasmid pALC (squares); pALC_*sprA2* (circles); pALC_*sprA2*^A53T,T54A^ (crosses); or pALC_*sprA2*^G52C^ (triangles). Induction was done after 2 h with 1 μM anhydrotetracycline (aTc).

We further investigated the *sprA2/sprA2_AS_* module to verify whether it encodes a functional type I TA system. To that aim, we cloned *sprA2* into pALC (28), allowing anhydrotetracycline-inducible expression, and cloned *sprA2_AS_* into high-copy-number pCN35 under its own promoter (29). We induced SprA2 expression by adding 1 μM aTc, then monitored *S. aureus* growth for 8 h (Figure 2A). While cells replicating both empty vectors exhibited typical bacterial growth (squares), growth of the *S. aureus* cells overexpressing only SprA2 was immediately arrested, with OD measurements decreasing by ∼50% over the next 6 h, suggesting cell death (circles). To some extent, this sudden arrest was relieved by the concomitant episomal expression of SprA2_AS_ (triangles). In this case, bacterial growth resumed after an apparent stasis of 2 h, finishing with a biomass close to that of the control strain. To further confirm that the decreased OD observed with SprA2 overexpression was due to bacterial death, serial dilutions of the respective cultures were plated without aTc (Figure 2B). Prior to induction, not much difference was observed between the three clones (left panel). However, most cells that overexpressed SprA2 did not grow on agar plates, indicating that they were not viable (right panel). Cell death was counterbalanced when SprA2_AS_ was overexpressed in pCN35, although complete suppression of toxicity could not be achieved. Similar experiments were performed with the *sprA1/sprA1_AS_* system (Supplementary Figure S2), known to have antibacterial activity. Here, SprA1 overexpression led to cell death which was fully relieved by SprA1_AS_ overexpression, suggesting that SprA2 RNA expression leads to stronger toxicity than does SprA1. To verify whether cell toxicity was due to the expression of a peptide rather than the SprA2 RNA itself, we tagged PepA2 with a FLAG sequence at its N-terminus. As described previously, the addition of a FLAG sequence usually decreases peptide toxicity, allowing for its expression to be monitored (17). Then, to avoid any potential repression of ^FLAG^PepA2 expression by endogenous SprA2_AS_, we deleted the entire putative type I TA locus in the Newman strain. We expressed SprA2-FLAG under its own promoter and in the presence or absence of SprA2_AS_ in Newman *ΔsprA2ΔsprA2_AS_* (Figure 3A and Supplementary Figure S3). Western blotting indicated that a peptide was expressed from *sprA2*, especially at OD_600 nm_ values of 1 and 2, and that the presence of SprA2_AS_ lowered peptide expression. Additionally, we collected the growth supernatant and found that a fraction of the ^FLAG^PepA2 was released in the extracellular medium, mostly at OD_600 nm_ of 6 and in the absence of SprA2_AS_ (Figure 3A). To prevent PepA2 expression, we made point mutations in *sprA2*, as shown in Figure 3B. Each mutated version was cloned into pALC and expression was induced with aTc (Figure 3C). Mutating the start codon (SprA2^A53T,T54A^) to prevent translation initiation did not inhibit growth. Since this absence of growth inhibition could be due to a substantial structural modification in SprA2 RNA rather than to a lack of PepA2 expression, we generated a point mutation just before the start codon that allows translation (SprA2^G52C^ in Figure 3B). This construct led to a sudden growth arrest similar to that observed previously, confirming that toxicity is dependent on PepA2 expression and that it is not due to the RNA. Altogether, our results indicate that *sprA2/sprA2_AS_* encodes a functional type I TA system, with *sprA2* triggering *S. aureus* death.

### PepA2 kills *Staphylococcus aureus* intracellularly and acts as an extracellular cytotoxin against human erythrocytes and polymorphonuclear neutrophils

**Figure 4.**
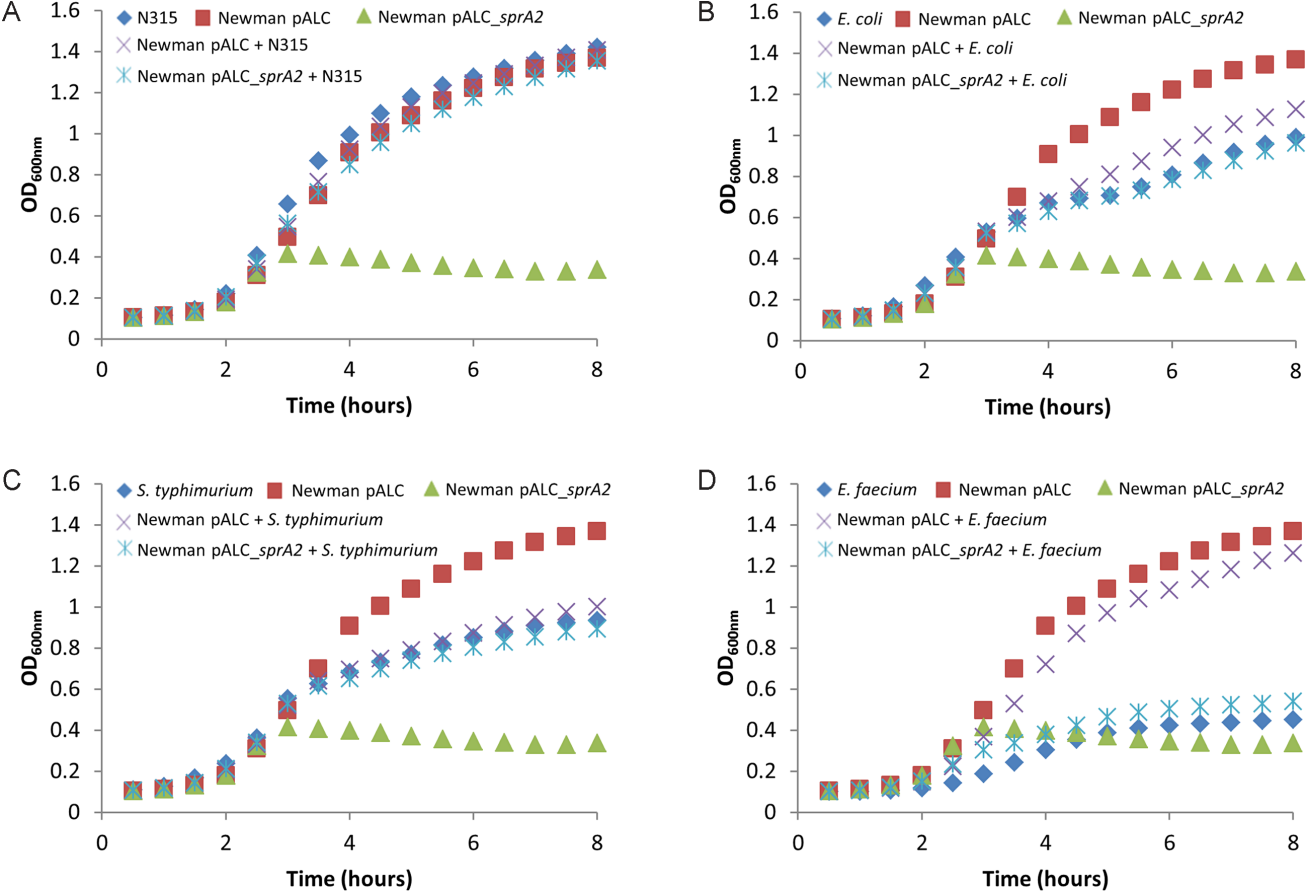
The SprA2 toxin does not kill other bacteria during co-cultures. (**A**) The effects of *sprA2* expression on the growth of *Staphylococcus aureus* N315. Shown are: *S. aureus* N315 (blue diamonds); *S. aureus* Newman pALC (red squares); Newman pALC_*sprA2* (green triangles); Newman pALC + N315 (purple crosses); and Newman pALC_*sprA2* + N315 (blue crosses). (**B**) The effects of *sprA2* expression on *Escherichia coli* growth. Shown are: *E. coli* EC101 (blue diamonds); *S. aureus* Newman pALC (red squares); Newman pALC_*sprA2* (green triangles); Newman pALC + *E. coli* (purple crosses); and Newman pALC_*sprA2* + *E. coli* (blue crosses). (**C**) The effects of SprA2 expression on *Salmonella typhimurium* growth. Shown are: *S. typhimurium* (blue diamonds); *S. aureus* Newman pALC (red squares); Newman pALC_*sprA2* (green triangles), Newman pALC + *S. typhimurium* (purple crosses); and Newman pALC_*sprA2* + *S. typhimurium* (blue crosses). (**D**) The effects of SprA2 expression on *Enterococcus faecium* growth. Shown are: *E. faecium* Aus004 (blue diamonds); *S. aureus* Newman pALC (red squares); Newman pALC_*sprA2* (green triangles); Newman pALC + *E. faecium* (purple crosses); and Newman pALC_*sprA2* + *E. faecium* (blue crosses). Growth was conducted in a Biotek instrument and 1 μM aTc was added after 2.5 h. The data presented are the mean of three independent experiments performed in triplicate. To improve readability, the standard deviations (which never exceeded 0.057) were omitted.

**Figure 5.**
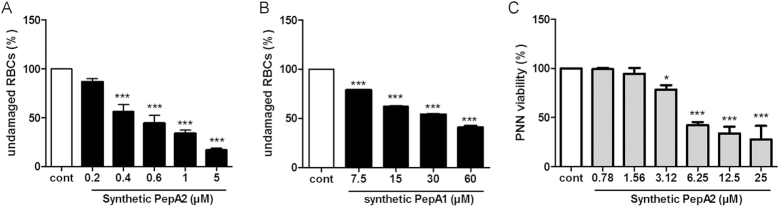
SprA2 is cytotoxic against human erythrocytes and polymorphonuclear neutrophils (PMNs) from blood donors. (**A**) Hemolytic activity of synthetic SprA2-encoded peptides on human erythrocytes (RBCs). (**B**) Hemolytic activity of synthetic SprA1-encoded peptides on human RBCs. (**C**) Cytotoxicity of synthetic SprA2-encoded peptides on PMN was estimated by flow cytometry using propidium iodide staining. The data are the mean of at least three independent biological experiments performed in triplicate. *P< 0.05; ***P< 0.001.

We pursued our investigations on cell toxicity, conducting co-cultures with both Gram-positive and Gram-negative bacteria. The bacteria were mixed at equal optical densities, and growth was followed in a Biotek instrument. After 2.5 h growth, cultures were subjected to 1 μM aTc to promote SprA2 expression (Figure 4). For all co-cultures tested, we could discriminate the species based on the use of selective or elective media (Supplementary Figure S4). When mixed with erythromycin-resistant *S. aureus* N315, *S. aureus* Newman growth stopped upon SprA2 induction, but N315 continued growing, which indicates that the SprA2 toxin acts intracellularly and cannot kill *S. aureus* from the outside (Figure 4A and Supplementary Figure S4). Conversely, no significant growth differences were observed in cultures not challenged with aTc (Supplementary Figure S5). We next tested the effects of the toxin on the growth of kanamycin-resistant *Escherichia coli* EC101 (Figure 4B), *Salmonella typhimurium* (Figure 4C), and *Enterococcus faecium* Aus004 (Figure 4D). In all of the tests, PepA2 overexpression arrested the growth of *S. aureus* Newman pALC_*sprA2*, but not of the other species. To confirm that extracellular PepA2 was inefficient for bacterial killing, we chemically synthesized and purified PepA2. Synthetic PepA2 was added to bacterial cultures at various concentrations to determine the minimum inhibitory concentration (MIC). PepA2 did not substantially inhibit the growths of *S. aureus, E. coli* or *S. typhimurium*, and only weakly inhibited *E. faecium* (Supplementary Figure S6). Furthermore, SprA2 overexpression in *E. coli* inhibited growth, indicating that PepA2 can also target Gram-negative bacteria from the inside, although not from the outside (Supplementary Figure S7). We further characterized synthetic PepA2’s toxic activity by adding it extracellularly to human erythrocytes and to polymorphonuclear neutrophils (PMNs) from different blood donors (Figure 5). PepA2 was highly hemolytic, with 50% hemolysis (H_50_) reached with ∼0.4 μM PepA2 (Figure 5A). On the other hand, chemical synthesis of PepA1 and its subsequent use for hemolysis revealed that more than 30 μM of this peptide was needed to obtain the same effect on human red blood cells (Figure 5B). We next tested the cytotoxic effect of PepA2 against PMNs (Figure 5C). PepA2 was also cytotoxic in this case, with an IC_50_ around 6 μM. Taken together, our data indicate that PepA2 induces bacterial cell death internally and, when it is released in the medium it is cytotoxic against human host cells, but not efficiently antibacterial.

### The non-overlapping 5′ end of SprA2_AS_ regulates toxin production and prevents PepA2 translation initiation

**Figure 6.**
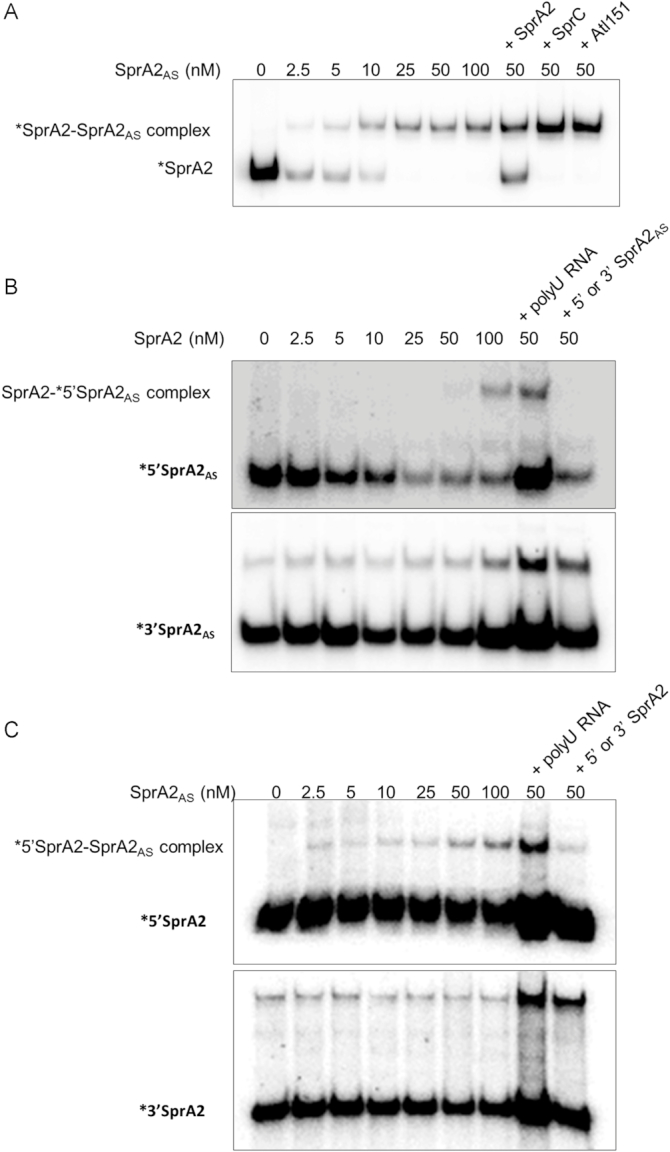
The non-overlapping domains of the SprA2 and SprA2_AS_ RNAs interact with each other. (**A**) Native gel shift assays of purified labelled SprA2 with increasing amounts of unlabelled SprA2_AS_. To assess binding specificity, 500 nM unlabelled SprA2, SprC, and Atl were individually added to the complex. (**B**) Complex formation between 5′ or 3′ SprA2_AS_ and SprA2. To assess complex formation specificity, 200 nM truncated SprA2_AS_ and 2 μM polyuridine (polyU) RNAs were added. (**C**) Complex formation between 5′ or 3′ SprA2 and SprA2_AS_. To assess complex specificity, 200 nM truncated SprA2 and 2 μM of polyU RNAs were added. Labelled RNAs (10 nM in each gel) are indicated with asterisks. The upper bands present in the lower panels of B and C represent unspecific bands as it is also detected in the absence of a potential partner (lanes 1). The data illustrate one representative experiment from three independent replicates.

To investigate the regulation between the toxin and antitoxin RNA in the *sprA2/sprA2_AS_* type I TA system, we began with gel shift assays. We mixed labelled SprA2 with increasing amounts of SprA2_AS_ and found that the RNAs interact to form a complex (Figure 6A). This indicated a direct RNA-RNA interaction, without the need of a third party. The apparent binding constant between the two is ∼13 nM, and the complex formation specificity was assessed by adding a 50-fold excess of various unlabelled RNAs (Figure 6A, last three lanes). While an excess of unlabelled SprA2 displaced the complex, the addition of unrelated Srn_3610_SprC sRNA (30) or a fragment of autolysin mRNA did modify the binding of SprA2_AS_ onto SprA2 RNA, implying specificity. We then went on to examine whether the 5′ non-overlapping region of SprA2_AS_ was responsible for the interaction between the two RNAs (Figure 6B and C). For this, we transcribed 5′ and 3′ moieties of SprA2 and SprA2_AS_ (Supplementary Figure S8). As shown in Figure 6B, only the 5′ half of SprA2_AS_ formed a specific complex with SprA2. Similarly, unlabelled SprA2_AS_ only slowed the migration of 5′ SprA2 (Figure 6C). These results indicate that the 5′ non-overlapping domains of both RNAs interact together *in vitro*, and demonstrate that although they are transcribed from the same locus (Figure 1A and C), SprA2_AS_ regulates SprA2 expression in *trans*.

**Figure 7.**
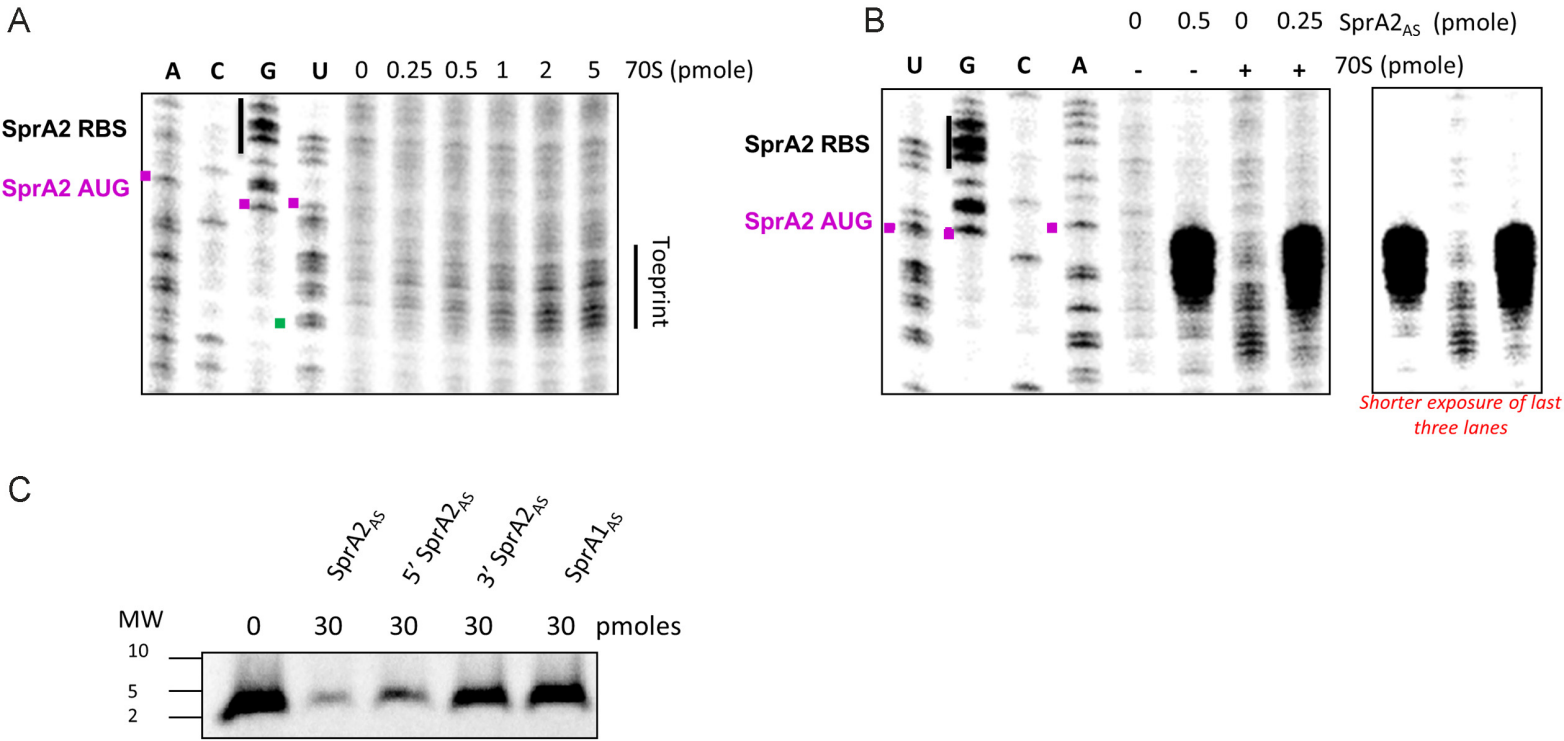
SprA2_AS_ prevents SprA2 translation by using its non-overlapping RNA domain. (**A**) Toeprinting assays of 0.25 pmol SprA2 in the presence of increasing amounts of purified *E. coli* 70S ribosomes revealed toeprints from position U71. The A, C, G and U lanes represent the SprA2 sequencing lanes. (**B**) Toeprinting assays of 0.25 pmol SprA2 in the presence of SprA2_AS_ and/or purified *E. coli* 70S ribosomes. The toeprint initially observed from position U71 decreased in the presence of the antitoxin. However, a strong pause appears at position A64 upon SprA2_AS_ addition, suggesting a conformational change in SprA2. In the right panel, a shorter exposure is provided to show the effect of SprA2_AS_ on the toeprint. (**C**) *In vitro* translation assay of 20 pmol SprA2 in the presence of 30 pmol of SprA2_AS_, 5′ SprA2_AS_ RNA, 3′ SprA2_AS_, or SprA1_AS_ (lanes 1–5, respectively). SprA2 encodes PepA2, a peptide weighing ∼4 kDa.

As presented in Figure 1C, this *trans*-regulation is predicted to mask the SprA2 ribosomal binding site (RBS). We therefore conducted toeprinting assays to see whether SprA2_AS_ would compete with ribosome binding onto SprA2. We began by looking at SprA2 in the presence of increasing amounts of purified *E. coli* ribosomes (Figure 7A). The assays showed that SprA2 recruits ribosomes to form translation initiation complexes in the presence of the initiator tRNA^fMet^. From 1 pmol of 70S ribosomes, the toeprint was detected approximately 14 nucleotides downstream from the predicted initiation codon (Figure 7A, lanes 9 and 10). We then tested whether the antitoxin SprA2_AS_ could prevent ribosomal loading onto SprA2 (Figure 7B), so the antitoxin and/or ribosomes were mixed with SprA2. A strong pause was detected starting at position A72 during the reverse transcription of SprA2 in the presence of its RNA antitoxin (Figure 7B, lane 6), suggesting that the association between the two RNAs induces a structural modification in SprA2 which prevents primer extension. The addition of both SprA2_AS_ and ribosomes resulted in an absence of ribosomal loading, whereas the pause due to the antitoxin's presence was still detected (Figure 7B, lane 8 and right panel). This indicated that SprA2_AS_ prevents ribosomal loading onto SprA2 and therefore PepA2 translation. To support this, we performed *in vitro* translation of SprA2 in the presence of either the full-length RNA antitoxin or its 5′ or 3′ truncated versions (Figure 7C). Whereas SprA2 translation did occur *in vitro* (lane 1), it was inhibited by both SprA2_AS_ and by the truncated version containing the main binding domain (lanes 2 and 3). Conversely, the 3′ part of the antitoxin did not significantly inhibit translation (lane 4). Also, the addition of non-cognate antitoxin SprA1_AS_ did not prevent translation *in vitro* (lane 5), suggesting that members of the SprA TA systems are specific to each other. SprA2_AS_ thus regulates the expression of SprA2, at least at the translational level, and its non-overlapping 5′ domain binds SprA2 at the RBS to prevent ribosomal loading and PepA2 expression.

### The SprA2/SprA2_AS_ and SprA1/SprA1_AS_ pairs act independently, with no cross-regulations between the antitoxins to rescue cell toxicity

**Figure 8.**
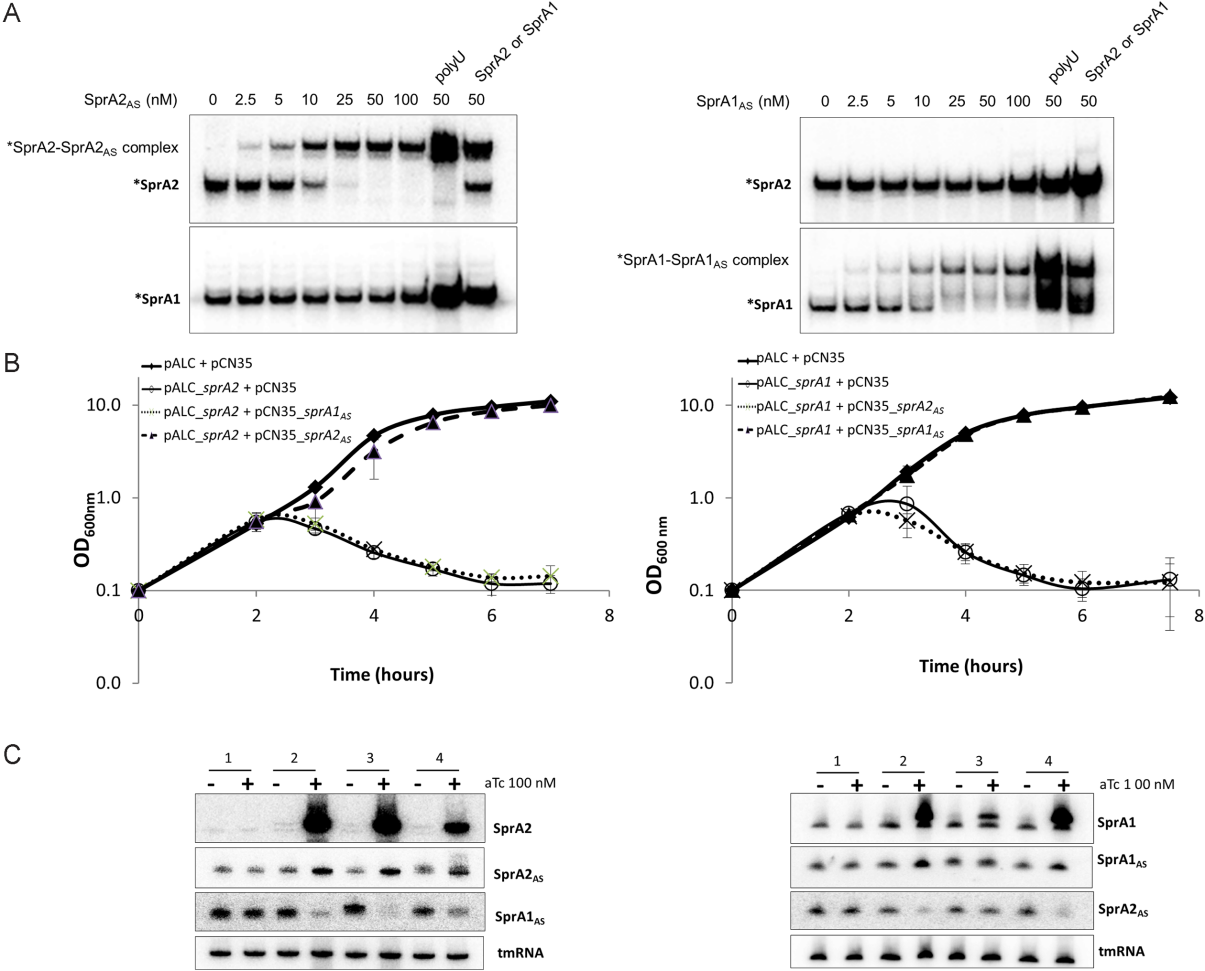
SprA2/SprA2_AS_ and SprA1/SprA1_AS_ pairs act independently. (**A**) Native gel shift assays of purified labelled SprA2 and SprA1 toxins with increasing amounts of (left) unlabelled SprA2_AS_ or (right) SprA1_AS_ antitoxins. To assess complex specificity, 200 nM of either toxin's RNA or 2 μM polyuridine (polyU) RNA was added. (**B**) Comparative growth curves to assess the antitoxin roles of SprA1_AS_ and SprA2_AS_. Cells were grown for 2 h, after which 100 nM anhydrotetracycline (aTc) was added to overexpress the Pep toxins. Shown are *Staphylococcus aureus* Newman strains containing (left) empty plasmids pALC + pCN35 (squares); pALC_*sprA2* + pCN35 (circles); pALC_*sprA2* + pCN35_*sprA1_AS_* (crosses); or pALC_*sprA2* + pCN35_*sprA2_AS_* (triangles); or (right) empty plasmids pALC + pCN35 (squares); pALC_*sprA1* + pCN35 (circles); pALC_*sprA1* + pCN35_*sprA1_AS_* (triangles); or pALC_*sprA1* + pCN35_*sprA2_AS_* (crosses). (**C**) RNA expression profiles after 30 min of aTc induction. Shown are *S. aureus* Newman strains containing: (left) pALC + pCN35; pALC_*sprA2* + pCN35; pALC_*sprA2* + pCN35_*sprA1_AS_*; and pALC_*sprA2* + pCN35_*sprA2_AS_*, listed as 1–4, respectively; and (right) pALC + pCN35; pALC_*sprA1* + pCN35; pALC_*sprA1* + pCN35_*sprA1_AS_*; and pALC_*sprA1* + pCN35_*sprA2_AS_*, listed as 1–4, respectively_._ tmRNA was used as the loading control.

These results revealed a regulation mechanism similar to that observed in the SprA1/SprA1_AS_ TA system (17). However, although both TA systems have nucleotide sequence similarities (Supplementary Figure S1), *in vitro* translation assays indicated that SprA1_AS_ cannot inhibit SprA2 translation. Therefore, we wondered whether the TA systems act independently, or if there is a cross-reactivity. First, we performed gel shift assays to demonstrate whether such cross interactions can occur *in vitro* (Figure 8A). When increasing amounts of unlabeled SprA2_AS_ RNA were added to labelled SprA2 or SprA1 RNA, RNA–RNA interactions were observed only between SprA2 and SprA2_AS_ (Figure 8A, left panel). Conversely, unlabelled SprA1_AS_ RNA only slowed the migration of labeled SprA1 (Figure 8A, right panel), indicating that both TA systems are specific to themselves *in vitro*. To support this, we tested the potential of each antitoxin to interact with their non-cognate toxins during bacterial culture. Genes coding the toxins were cloned into the aTc-inducible pALC vector, while the antitoxins were cloned into pCN35 under their own promoters (Figure 8B). Cell death due to SprA2 overexpression was not relieved by the presence of the SprA1_AS_ antitoxin (Figure 8B; crosses in left panel), consistent with the *in vitro* results. Similarly, SprA1 toxicity was relieved only when its SprA1_AS_ cognate antitoxin was expressed from pCN35 (Figure 8B, right panel). Total RNA extractions performed under these growth conditions confirmed that all of the TA module components were expressed, and that overexpression of SprA2 or SprA1 was indeed effective (Figure 8C). While the antitoxin RNA levels did increase when their cognate toxins were induced, the RNA levels of the other antitoxins went down (Figure 8C, conditions 2 to 4). Therefore, we monitored the transcript levels of another type I TA module (SprG1/SprF1) and of two regulatory RNAs, Srn_9340 and Srn_3610_SprC (Supplementary Figure S9). SprF1 and these regulatory RNAs have half-lives of 10, 5 and 20 min, respectively (25,30), and they all showed reduced transcript levels upon SprA1/2 induction. Conversely, the RNA levels of SprG1, which has a half-life of ∼2 h (20), did not decrease after SprA1/2 induction. This suggests that the decreased levels of RNAs with short or moderate half-lives are probably due to cell death, which favours rapid RNA degradation.

### SprA2 overexpression increases the stability of SprA2_AS_ RNA

**Figure 9.**
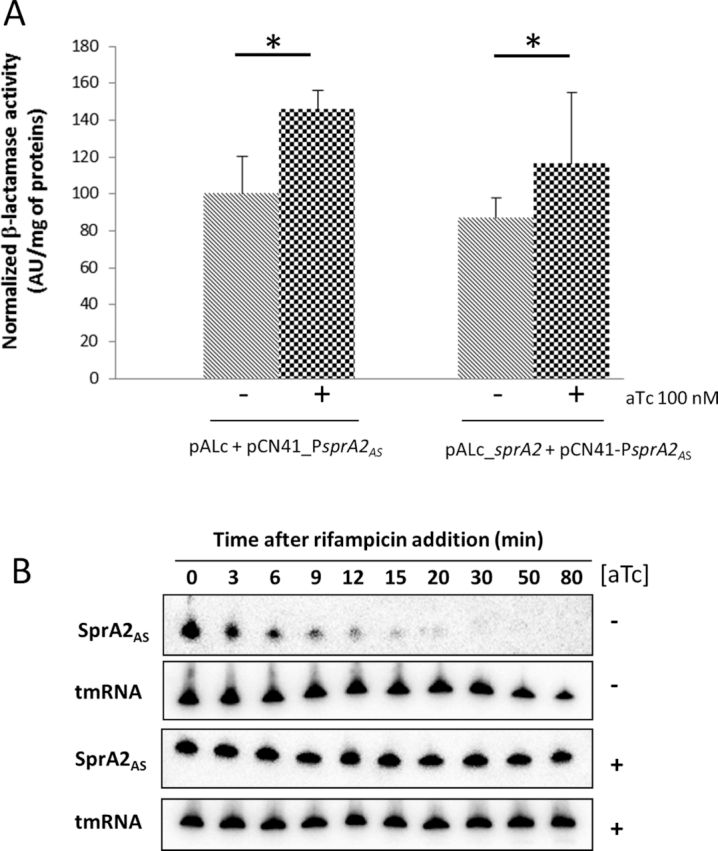
The presence of SprA2 affects SprA2_AS_ stability. (**A**) The effects of SprA2 on the transcriptional activity of the *SprA2_AS_* promoter (P*sprA2_AS_*). *S. aureus* Newman pALC and pALC_*sprA2* were co-transformed with pCN41_P*sprA2_AS_*. SprA2 expression was induced with 100 nM anhydrotetracycline (aTc) and ß-lactamase activity was measured after 30 min. For each lane, the activity was normalized by subtracting the background signal from the same strain in which pCN41c_P*sprA2_AS_* was replaced by the pCN41 empty vector (not shown). The data presented are the mean of three independent experiments performed in triplicate ± standard deviation. *P < 0.05 (Mann-Whitney U test). (**B**) The half-life of SprA2_AS_ increased when SprA2 was overexpressed. *S. aureus* Newman strain with pALC_*sprA2* + pCN35_*sprA2*_*AS*_was grown in brain heart diffusion, then SprA2 expression was induced by 100 nM aTc. After 30 min, 200 μg/ml rifampicin was added to the culture, and total RNAs extracted at different time intervals. tmRNA was used as an internal loading control. The data shown are from one representative experiment out of three performed.

Increased SprA2_AS_ antitoxin RNA in response to the overexpression of its cognate toxin suggested an effect at the levels or transcription and/or stability. To measure antitoxin expression, we cloned the *sprA2_AS_* promoter into pCN41, allowing transcriptional fusion with *blaZ*, which encodes β-lactamase. *S. aureus* Newman pCN41_P*sprA2*_AS_ was transformed with either pALC or pALC_SprA2 (Figure 9). A 1.3-fold increase in β–lactamase activity was measured when aTc was added to promote SprA2 expression. Similarly, when aTc was added to the strain co-transformed with pALC, β–lactamase activity increased ∼1.4-fold, indicating that the inducer itself weakly activates P*sprA2_AS_* in the absence of SprA2 overexpression. Conversely, no significant variations were detected between strains overexpressing SprA2 and the control, indicating that SprA2 does not directly or indirectly regulate the transcription of its antitoxin. We therefore decided to look at SprA2_AS_ RNA stability with or without SprA2 induction. To do this we used Newman pALC_*sprA2* + pCN35_*sprA2_AS_* (see Figure 8) and inhibited transcription with rifampicin (Figure 9B). While the half-life of SprA2_AS_ was around 5 min in the presence of endogenous SprA2, SprA2 induction increased its antitoxin's stability by ∼9-fold, with a half-life of around 45 min. SprA2 expression thus increases SprA2_AS_ stability, but not its transcriptional activity.

### Antitoxin expression levels are reduced under osmotic shock and stringent conditions

**Figure 10.**
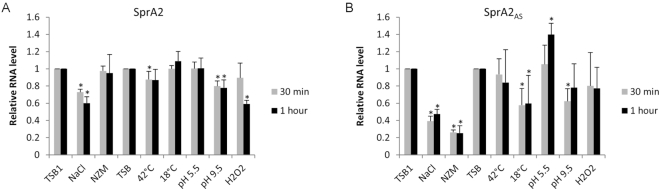
Expression of *SprA2* and *SprA2_AS_* monitored by northern blots under various stresses. *Staphylococcus aureus* Newman was cultured in tryptic soy broth (TSB) to an OD_600_ of 2, stress applied, then RNA extracted to monitor (**A**) SprA2 and (**B**) SprA2_AS_ transcript levels. Stress was induced either by centrifuging bacteria prior to resuspension in appropriate medium, or performed by directly adding the stressor to the growth medium. Pellets were resuspended in fresh TSB (TSB1), TSB supplemented with 1 M NaCl (mimicking osmotic stress), or in NZM medium (emulating a stringent response). Other stresses were induced by adding H_2_O_2_, HCl or NaOH, or by changing the temperature to 18 or 42°C. The TSB lane corresponds to cells maintained in TSB under normal conditions. The relative expression levels were calculated after quantification of northern blot bands using TSB1 or TSB as calibrators. For all experiments, tmRNA was used as an internal loading control. The data presented are the mean of three independent experiments. Statistical analysis was conducted using the one-tailed Mann–Whitney test; differences were considered statistically significant when *P* < 0.05.

The triggers involved in the expression and regulation of this newly described type I TA system are unknown. To explore this question, we applied different stresses, some mimicking the changes encountered by the bacteria during infection. SprA2 and SprA2_AS_ RNA levels were monitored by northern blots after 30 and 60 min stress, then compared with those produced without stress (Figure 10). The Mann-Whitney U test was used to locate statistically significant differences, with biologically relevant differences being anything over the defined cut-off value of 2. The SprA2 RNA levels were weakly affected by all conditions applied (Figure 10A), but although we measured variations, they were always lower than a two-fold change (i.e. a 1.7-fold decrease in 1 M NaCl after undergoing stress for 1 h). Conversely, after 30 or 60 min of stringent (NZM) or osmotic (NaCl) stress, SprA2_AS_ RNA levels significantly decreased (Figure 10B). In 1 M NaCl, antitoxin RNA was decreased more than two-fold, whereas it was lowered by around four in NZM medium. While the RNA levels were affected in these two cases, only a slight decrease (under the cut-off) in SprA2 transcript levels was measured in 1 M NaCl (Figure 10A). Other variations in antitoxin levels were lower than two-fold increases or decreases (Figure 10B). Altogether, these data indicate that *sprA2* and *sprA2_AS_* are differentially expressed in response to stressors, with antitoxin RNA levels reduced under stringent conditions and during osmotic shock. In addition, this reduction was not accompanied by a strong change in SprA2 transcript levels. Overall, this suggests that stringent conditions and elevated osmotic pressure could be triggers for promoting PepA2 toxin production.

## DISCUSSION

Toxin-antitoxin modules are widespread in bacteria and archaea (14,15). They are known to participate in various functions, including irreversible membrane damage, plasmid maintenance through post-segregational killing, cell death or stasis, global translation inhibition, and a recently discovered role in persistence (15,19,22,24). They are divided into six different types, with three described in *S. aureus* (15). To date, two type-I systems have been characterized in *S. aureus*: the SprA1/SprA1_AS_ and SprG1/SprF1 systems identified and expressed from pathogenicity islands (17,20).

In bacterial genomes, type I TA systems are commonly found in multiple copies. In this work, we describe the SprA2/SprA2_AS_ module, which has more than 70% nucleotide sequence identity with the SprA1/SprA1_AS_ TA system (7,13,17). We report here the second example of a type I TA system in which a *cis*-encoded antitoxin acts in *trans* with its cognate toxin to prevent ribosomal loading and thus the translation of a toxic peptide. We showed that the 5′ part of the antitoxin's RNA is sufficient to bind SprA2 RNA *in vitro*, while its 3′ moiety, sharing extensive complementary due to the overlapping regions, has only a minor effect on regulation. Indeed, no inhibition of SprA2 translation was detected with 30 pmol of 3′ SprA2_AS_, whereas strong inhibition was already observed using the same amount of 5′ SprA2_AS_ (Figure 7C). Moreover, SprA2_AS_ binds its target with high affinity: its apparent *K*_d_ of 13 nM is similar to the 15 nM observed for the SprA1/SprA1_AS_ TA system, and is slightly higher than the >1 nM value for SprG1/SprF1 (17,20). Our in-depth characterization of the mechanism involved indicates that SprA2 overexpression (and therefore PepA2 expression) has no effect on the activity of the P*sprA2_as_* promoter, but it increases the stability of the antitoxin by nine, presumably due to its titration by SprA2 and an enhanced stability of the complex.

In the present work, we investigated whether cross-regulation occurs between SprA1/SprA1_AS_ and SprA2/SprA2_AS_ type I TA modules. Instances of crosstalk are largely under documented (31,32). To our knowledge, so far crosstalk has only been reported in other bacteria for one type II TA system (33) and also elegantly between a type I and a type II TA system (34) and between a type II and type V TA system (35). Here, our *in vivo* and *in vitro* analyses showed that when it comes to toxicity regulation, the antitoxins involved are specific to their cognate modules. Moreover, we showed that the SprA2_AS_ antitoxin responds to an osmotic shock and to nutrient starvation, whereas previous studies conducted on the SprA1/SprA1_AS_ TA system revealed that PepA1 expression increased upon oxidative and acidic stresses due to a decrease in antitoxin levels (18). Both studies indicate that the key factor for allowing or repressing PepA1 or PepA2 expression is the corresponding antitoxin RNA level. PepA2 shares 50% amino acid identity with PepA1 (Supplementary Figure S1). Both of the PepA1 and PepA2 N-terminal domains are rich in hydrophobic aliphatic amino acids that confer hydrophobicity and hemolytic potencies (26,36,37). Such hydrophobic content is also encountered in SprG1, another staphylococcal type I toxin (20) or also in the phenol-soluble modulins (38). Many type I TA systems form pores in bacterial membranes, inducing bacterial death (16,18,38,39). However, in our study, PepA1 and PepA2 exhibit different features. While PepA1 has both antimicrobial and hemolytic activity (17,18,26), our data indicates that PepA2 is mostly cytotoxic, rather than acting very strongly against either Gram-positive and Gram-negative bacteria. PepA2 is about 10 times more effective on human erythrocytes than PepA1 (Figure 5). Our results suggest that PepA2 has a serious potential to damage eukaryotic membranes compared to bacterial membranes, and may belong to *Staphylococcus aureus* virulence arsenal, along with other leukocidins such as the phenol-soluble modulins (40).

Overall, because of the specificity of each antitoxin to a single RNA and their differences in toxin targets, effectiveness, and responses to various environmental stimuli, we raise the question of their redundancies. Redundancy within TA systems is usually attributed to the fact that deletion of a single TA system was insufficient to confer a significant phenotype (41). TA systems were originally described in post-segregational killing mechanism and to mediate programmed cell death (42), therefore being efficient killers. It was then demonstrated that they could induce stasis (42), and several recent studies have called attention to their role in commitment to persistence through fluctuations in the alarmone (p)ppGpp involved in the stringent response (41). Therefore, TA systems (including type I) have a broad spectrum of action (14,16), and are sometimes difficult to link to a phenotype (43). Here, our data do not pinpoint a specific role for the SprA2/SprA2_AS_ module in bacterial stasis or persistence. Contrary to (42), overexpression of SprA2 led to a sudden growth arrest followed by decreased absorbance measures. This suggests cell death, a conclusion supported after spreading cells on agar plates in the absence of the inducer. Expression levels measured under different stress conditions allowed us to identify a drop in SprA2_AS_ RNA levels in the case of hyperosmotic conditions and during nutrient starvation, which promotes a stringent response. A functional link between SprA2/SprA2_AS_ and (p)ppGpp has not yet been investigated. However, our data does suggest that each TA system has dedicated effectors and roles and must therefore participate in the adaptive response to environmental changes (44–46), possibly including those encountered when in contact with the host during infection or colonization. Indeed, there is growing evidence in recent publications for TA systems that are not linked to persister cell formation (45). In *S. aureus*, the discovery and characterization of type I TA systems is limited to a few studies (8,17,20), all of which involve cell death. In other bacterial and clinical pathogens, type I TA modules have various mechanisms of action, functions, and triggers (14,16,24,47). These include plasmid maintenance, regulation of the SOS response, abortive phage infection, and biofilm formation (48–52). Conversely, more is known about staphylococcal type II TA systems (43). A recent article demonstrated that persister levels were unaffected when all known type II TA systems were knocked out (53). In that study, the authors showed that persister production is critically dependent on the drop in intracellular ATP, and this might be the case for all Gram-positive bacteria. Additionally, studies on the *S. aureus* type II system MazEF pinpointed a possible regulatory relationship with staphylococcal pathogenicity (54). Similarly, the PemIKSa TA system's participation in staphylococcal virulence regulation could be linked to an altered translation of a large set of genes that promote virulence factor expression (55). Finally, the physiological role of SavRS, a novel type II *S. aureus* TA system, was recently described (56). The deletion of this system increased hemolytic activity and pathogenicity in a mouse subcutaneous abscess model. Additionally, the authors showed that the TA system could repress virulence gene expression by direct binding. Ongoing efforts to characterize the different SprFs/SprGs type I TA modules support the hypothesis that each TA system has specific roles. SprG1 is an efficient killer (20), whereas some of the newly characterized SprG toxins induce bacterial stasis and are therefore probably involved in persistence (57). Additionally, studies on the SprGs/SprFs system revealed that they respond to different triggers, and that overexpression of one component does not increase the cognate partner's stability but reduces the cognate's transcript levels. Altogether, recent progress in the study and characterization of staphylococcal type I TA systems, suggest that their biological roles, mode of actions, and redundancies may be reconsidered. Our findings provide novel insights into the roles and functions of such a system. They suggest that, *in vivo, S. aureus* may specifically control PepA2 expression to induce cytotoxicity when it is necessary, especially during infection. A tight control of PepA2 expression would therefore be needed to avoid bacterial death internally. Alternately, some clones might behave altruistically by producing PepA2 and undergoing cell death while promoting cytotoxicity, thus guaranteeing the success and fitness of the community.

## Supplementary Material

Supplementary Data

## References

[1] Anstead G.M., Cadena J., Javeri H. Treatment of infections due to resistant Staphylococcus aureus. Methods Mol. Biol. 2014; 1085:259–309. 24085702 10.1007/978-1-62703-664-1_16

[2] Zecconi A., Scali F. Staphylococcus aureus virulence factors in evasion from innate immune defenses in human and animal diseases. Immunol. Lett. 2013; 150:12–22. 23376548 10.1016/j.imlet.2013.01.004

[3] Felden B., Vandenesch F., Bouloc P., Romby P. The Staphylococcus aureus RNome and its commitment to virulence. PLoS Pathog. 2011; 7:e1002006. 21423670 10.1371/journal.ppat.1002006 PMC3053349

[4] Caldelari I., Chao Y., Romby P., Vogel J. RNA-mediated regulation in pathogenic bacteria. Cold Spring Harb. Perspect. Med. 2013; 3:a010298. 24003243 10.1101/cshperspect.a010298 PMC3753719

[5] Fechter P., Caldelari I., Lioliou E., Romby P. Novel aspects of RNA regulation in *Staphylococcus aureus*. FEBS Lett. 2014; 588:2523–2529. 24873876 10.1016/j.febslet.2014.05.037

[6] Wagner E.G., Romby P. Small RNAs in bacteria and archaea: who they are, what they do, and how they do it. Adv. Genet. 2015; 90:133–208. 26296935 10.1016/bs.adgen.2015.05.001

[7] Sassi M., Augagneur Y., Mauro T., Ivain L., Chabelskaya S., Hallier M., Sallou O., Felden B. SRD: a Staphylococcus regulatory RNA database. RNA. 2015; 21:1005–1017. 25805861 10.1261/rna.049346.114 PMC4408781

[8] Beaume M., Hernandez D., Farinelli L., Deluen C., Linder P., Gaspin C., Romby P., Schrenzel J., Francois P. Cartography of methicillin-resistant S. aureus transcripts: detection, orientation and temporal expression during growth phase and stress conditions. PLoS One. 2010; 5:e10725. 20505759 10.1371/journal.pone.0010725 PMC2873960

[9] Bronsard J., Pascreau G., Sassi M., Mauro T., Augagneur Y., Felden B. sRNA and cis-antisense sRNA identification in Staphylococcus aureus highlights an unusual sRNA gene cluster with one encoding a secreted peptide. Sci. Rep. 2017; 7:4565. 28676719 10.1038/s41598-017-04786-3 PMC5496865

[10] Howden B.P., Beaume M., Harrison P.F., Hernandez D., Schrenzel J., Seemann T., Francois P., Stinear T.P. Analysis of the small RNA transcriptional response in multidrug-resistant Staphylococcus aureus after antimicrobial exposure. Antimicrob. Agents Chemother. 2013; 57:3864–3874. 23733475 10.1128/AAC.00263-13 PMC3719707

[11] Lasa I., Toledo-Arana A., Dobin A., Villanueva M., de los Mozos I.R., Vergara-Irigaray M., Segura V., Fagegaltier D., Penades J.R., Valle J. Genome-wide antisense transcription drives mRNA processing in bacteria. Proc. Natl. Acad. Sci. U.S.A. 2011; 108:20172–20177. 22123973 10.1073/pnas.1113521108 PMC3250193

[12] Liu W., Rochat T., Toffano-Nioche C., Le Lam T.N., Bouloc P., Morvan C. Assessment of Bona Fide sRNAs in Staphylococcus aureus. Front. Microbiol. 2018; 9:228. 29515534 10.3389/fmicb.2018.00228 PMC5826253

[13] Pichon C., Felden B. Small RNA genes expressed from Staphylococcus aureus genomic and pathogenicity islands with specific expression among pathogenic strains. Proc. Natl. Acad. Sci. U.S.A. 2005; 102:14249–14254. 16183745 10.1073/pnas.0503838102 PMC1242290

[14] Fernandez-Garcia L., Blasco L., Lopez M., Bou G., Garcia-Contreras R., Wood T., Tomas M. Toxin-Antitoxin systems in clinical pathogens. Toxins (Basel). 2016; 8:227. 10.3390/toxins8070227 PMC4963858 27447671

[15] Schuster C.F., Bertram R. Toxin-Antitoxin Systems of Staphylococcus aureus. Toxins (Basel). 2016; 8:140. 10.3390/toxins8050140 PMC4885055 27164142

[16] Brantl S., Jahn N. sRNAs in bacterial type I and type III toxin–antitoxin systems. FEMS Microbiol. Rev. 2015; 39:413–427. 25808661 10.1093/femsre/fuv003

[17] Sayed N., Jousselin A., Felden B. A cis-antisense RNA acts in trans in Staphylococcus aureus to control translation of a human cytolytic peptide. Nat. Struct. Mol. Biol. 2012; 19:105–112. 10.1038/nsmb.2193 22198463

[18] Sayed N., Nonin-Lecomte S., Rety S., Felden B. Functional and structural insights of a Staphylococcus aureus apoptotic-like membrane peptide from a toxin–antitoxin module. J. Biol. Chem. 2012; 287:43454–43463. 23129767 10.1074/jbc.M112.402693 PMC3527932

[19] Brielle R., Pinel-Marie M.L., Felden B. Linking bacterial type I toxins with their actions. Curr. Opin. Microbiol. 2016; 30:114–121. 26874964 10.1016/j.mib.2016.01.009

[20] Pinel-Marie M.L., Brielle R., Felden B. Dual toxic-peptide-coding Staphylococcus aureus RNA under antisense regulation targets host cells and bacterial rivals unequally. Cell Rep. 2014; 7:424–435. 24703849 10.1016/j.celrep.2014.03.012

[21] Fozo E.M., Hemm M.R., Storz G. Small toxic proteins and the antisense RNAs that repress them. Microbiol. Mol. Biol. Rev. 2008; 72:579–589. 19052321 10.1128/MMBR.00025-08 PMC2593563

[22] Balaban N.Q., Gerdes K., Lewis K., McKinney J.D. A problem of persistence: still more questions than answers. Nat. Rev. Microbiol. 2013; 11:587–591. 24020075 10.1038/nrmicro3076

[23] Wagner E.G., Brantl S. Kissing and RNA stability in antisense control of plasmid replication. Trends Biochem. Sci. 1998; 23:451–454. 9868360 10.1016/s0968-0004(98)01322-x

[24] Harms A., Brodersen D.E., Mitarai N., Gerdes K. Toxins, targets, and triggers: an overview of toxin-antitoxin biology. Mol. Cell. 2018; 70:768–784. 29398446 10.1016/j.molcel.2018.01.003

[25] Mauro T., Rouillon A., Felden B. Insights into the regulation of small RNA expression: SarA represses the expression of two sRNAs in Staphylococcus aureus. Nucleic Acids Res. 2016; 44:10186–10200. 27596601 10.1093/nar/gkw777 PMC5137438

[26] Solecki O., Mosbah A., Baudy Floc’h M., Felden B. Converting a Staphylococcus aureus toxin into effective cyclic pseudopeptide antibiotics. Chem. Biol. 2015; 22:329–335. 25728268 10.1016/j.chembiol.2014.12.016

[27] Busch A., Richter A.S., Backofen R. IntaRNA: efficient prediction of bacterial sRNA targets incorporating target site accessibility and seed regions. Bioinformatics. 2008; 24:2849–2856. 18940824 10.1093/bioinformatics/btn544 PMC2639303

[28] Helle L., Kull M., Mayer S., Marincola G., Zelder M.E., Goerke C., Wolz C., Bertram R. Vectors for improved Tet repressor-dependent gradual gene induction or silencing in Staphylococcus aureus. Microbiology. 2011; 157:3314–3323. 21921101 10.1099/mic.0.052548-0

[29] Charpentier E., Anton A.I., Barry P., Alfonso B., Fang Y., Novick R.P. Novel cassette-based shuttle vector system for gram-positive bacteria. Appl. Environ. Microbiol. 2004; 70:6076–6085. 15466553 10.1128/AEM.70.10.6076-6085.2004 PMC522135

[30] Le Pabic H., Germain-Amiot N., Bordeau V., Felden B. A bacterial regulatory RNA attenuates virulence, spread and human host cell phagocytosis. Nucleic Acids Res. 2015; 43:9232–9248. 26240382 10.1093/nar/gkv783 PMC4627067

[31] Iqbal N., Guerout A.M., Krin E., Le Roux F., Mazel D. Comprehensive functional analysis of the 18 vibrio cholerae N16961 Toxin-Antitoxin systems substantiates their role in stabilizing the superintegron. J. Bacteriol. 2015; 197:2150–2159. 25897030 10.1128/JB.00108-15 PMC4455273

[32] Yoshizumi S., Zhang Y., Yamaguchi Y., Chen L., Kreiswirth B.N., Inouye M. Staphylococcus aureus YoeB homologues inhibit translation initiation. J. Bacteriol. 2009; 191:5868–5872. 19581360 10.1128/JB.00623-09 PMC2737954

[33] Walling L.R., Butler J.S. Structural determinants for antitoxin identity and insulation of cross talk between homologous toxin-antitoxin systems. J. Bacteriol. 2016; 198:3287–3295. 27672196 10.1128/JB.00529-16 PMC5116932

[34] Wessner F., Lacoux C., Goeders N., Fouquier d’Herouel A., Matos R., Serror P., Van Melderen L., Repoila F. Regulatory crosstalk between type I and type II toxin–antitoxin systems in the human pathogen Enterococcus faecalis. RNA Biol. 2015; 12:1099–1108. 26305399 10.1080/15476286.2015.1084465 PMC4829291

[35] Wang X., Lord D.M., Hong S.H., Peti W., Benedik M.J., Page R., Wood T.K. Type II toxin/antitoxin MqsR/MqsA controls type V toxin/antitoxin GhoT/GhoS. Environ. Microbiol. 2013; 15:1734–1744. 23289863 10.1111/1462-2920.12063 PMC3620836

[36] Chaudhary K., Kumar R., Singh S., Tuknait A., Gautam A., Mathur D., Anand P., Varshney G.C., Raghava G.P. A web server and mobile app for computing hemolytic potency of peptides. Sci. Rep. 2016; 6:22843. 26953092 10.1038/srep22843 PMC4782144

[37] Chen Y., Guarnieri M.T., Vasil A.I., Vasil M.L., Mant C.T., Hodges R.S. Role of peptide hydrophobicity in the mechanism of action of alpha-helical antimicrobial peptides. Antimicrob. Agents Chemother. 2007; 51:1398–1406. 17158938 10.1128/AAC.00925-06 PMC1855469

[38] Brantl S. Bacterial type I toxin–antitoxin systems. RNA Biology. 2012; 9:1488–1490. 23324552 10.4161/rna.23045

[39] Gurnev P.A., Ortenberg R., Dorr T., Lewis K., Bezrukov S.M. Persister-promoting bacterial toxin TisB produces anion-selective pores in planar lipid bilayers. FEBS Lett. 2012; 586:2529–2534. 22728134 10.1016/j.febslet.2012.06.021 PMC3498054

[40] Wang R., Braughton K.R., Kretschmer D., Bach T.H., Queck S.Y., Li M., Kennedy A.D., Dorward D.W., Klebanoff S.J., Peschel A. Identification of novel cytolytic peptides as key virulence determinants for community-associated MRSA. Nat. Med. 2007; 13:1510–1514. 17994102 10.1038/nm1656

[41] Tian C., Semsey S., Mitarai N. Synchronized switching of multiple toxin–antitoxin modules by (p)ppGpp fluctuation. Nucleic Acids Res. 2017; 45:8180–8189. 28854732 10.1093/nar/gkx552 PMC5737467

[42] Pedersen K., Christensen S.K., Gerdes K. Rapid induction and reversal of a bacteriostatic condition by controlled expression of toxins and antitoxins. Mol. Microbiol. 2002; 45:501–510. 12123459 10.1046/j.1365-2958.2002.03027.x

[43] Sierra R., Viollier P., Renzoni A. Linking toxin–antitoxin systems with phenotypes: a Staphylococcus aureus viewpoint. Biochim. Biophys. ActaGene Regulatory Mechanisms. 2018; doi:10.1016/j.bbagrm.2018.07.009. 10.1016/j.bbagrm.2018.07.009 30056132

[44] Bukowski M., Rojowska A., Wladyka B. Prokaryotic toxin–antitoxin systems–the role in bacterial physiology and application in molecular biology. Acta Biochim. Pol. 2011; 58:1–9. 21394325

[45] Curtis T.D., Takeuchi I., Gram L., Knudsen G.M. The influence of the toxin/antitoxin mazEF on growth and survival of Listeria monocytogenes under stress. Toxins (Basel). 2017; 9:31. 10.3390/toxins9010031 PMC5308263 28098783

[46] Gerdes K., Christensen S.K., Lobner-Olesen A. Prokaryotic toxin–antitoxin stress response loci. Nat. Rev. Microbiol. 2005; 3:371–382. 15864262 10.1038/nrmicro1147

[47] Jahn N., Brantl S. One antitoxin–two functions: SR4 controls toxin mRNA decay and translation. Nucleic Acids Res. 2013; 41:9870–9880. 23969414 10.1093/nar/gkt735 PMC3834814

[48] Patel S., Weaver K.E. Addiction toxin Fst has unique effects on chromosome segregation and cell division in Enterococcus faecalis and Bacillus subtilis. J. Bacteriol. 2006; 188:5374–5384. 16855226 10.1128/JB.00513-06 PMC1540048

[49] Vogel J., Argaman L., Wagner E.G., Altuvia S. The small RNA IstR inhibits synthesis of an SOS-induced toxic peptide. Curr. Biol. 2004; 14:2271–2276. 15620655 10.1016/j.cub.2004.12.003

[50] Kawano M., Aravind L., Storz G. An antisense RNA controls synthesis of an SOS-induced toxin evolved from an antitoxin. Mol. Microbiol. 2007; 64:738–754. 17462020 10.1111/j.1365-2958.2007.05688.x PMC1891008

[51] Pecota D.C., Wood T.K. Exclusion of T4 phage by the hok/sok killer locus from plasmid R1. J. Bacteriol. 1996; 178:2044–2050. 8606182 10.1128/jb.178.7.2044-2050.1996 PMC177903

[52] Domka J., Lee J., Bansal T., Wood T.K. Temporal gene-expression in Escherichia coli K-12 biofilms. Environ. Microbiol. 2007; 9:332–346. 17222132 10.1111/j.1462-2920.2006.01143.x

[53] Conlon B.P., Rowe S.E., Gandt A.B., Nuxoll A.S., Donegan N.P., Zalis E.A., Clair G., Adkins J.N., Cheung A.L., Lewis K. Persister formation in Staphylococcus aureus is associated with ATP depletion. Nat. Microbiol. 2016; 1:16051. 10.1038/nmicrobiol.2016.51 PMC4932909 27572649

[54] Zhu L., Inoue K., Yoshizumi S., Kobayashi H., Zhang Y., Ouyang M., Kato F., Sugai M., Inouye M. Staphylococcus aureus MazF specifically cleaves a pentad sequence, UACAU, which is unusually abundant in the mRNA for pathogenic adhesive factor SraP. J. Bacteriol. 2009; 191:3248–3255. 19251861 10.1128/JB.01815-08 PMC2687152

[55] Bukowski M., Lyzen R., Helbin W.M., Bonar E., Szalewska-Palasz A., Wegrzyn G., Dubin G., Dubin A., Wladyka B. A regulatory role for Staphylococcus aureus toxin–antitoxin system PemIKSa. Nat. Commun. 2013; 4:2012. 23774061 10.1038/ncomms3012

[56] Wen W., Liu B., Xue L., Zhu Z., Niu L., Sun B. Autoregulation and virulence control by the toxin-antitoxin system SavRS in Staphylococcus aureus. Infect. Immun. 2018; 86:e00032–18. 29440365 10.1128/IAI.00032-18 PMC5913840

[57] Riffaud C., Pinel-Marie M.L., Pascreau G., Felden B. Functionality and cross-regulation of the four SprG/SprF type I toxin–antitoxin systems in *Staphylococcus aureus*. Nucleic Acids Res. 2018; doi:10.1093/nar/gky1256. 10.1093/nar/gky1256 PMC6393307 30551143

[58] Baba T., Bae T., Schneewind O., Takeuchi F., Hiramatsu K. Genome sequence of Staphylococcus aureus strain Newman and comparative analysis of staphylococcal genomes: polymorphism and evolution of two major pathogenicity islands. J. Bacteriol. 2008; 190:300–310. 17951380 10.1128/JB.01000-07 PMC2223734

[59] Kuroda M., Ohta T., Uchiyama I., Baba T., Yuzawa H., Kobayashi I., Cui L., Oguchi A., Aoki K., Nagai Y. Whole genome sequencing of meticillin-resistant *Staphylococcus aureus*. Lancet. 2001; 357:1225–1240. 11418146 10.1016/s0140-6736(00)04403-2

[60] Law J., Buist G., Haandrikman A., Kok J., Venema G., Leenhouts K. A system to generate chromosomal mutations in Lactococcus lactis which allows fast analysis of targeted genes. J. Bacteriol. 1995; 177:7011–7018. 8522504 10.1128/jb.177.24.7011-7018.1995 PMC177576

[61] McClelland M., Sanderson K.E., Spieth J., Clifton S.W., Latreille P., Courtney L., Porwollik S., Ali J., Dante M., Du F. Complete genome sequence of Salmonella enterica serovar Typhimurium LT2. Nature. 2001; 413:852–856. 11677609 10.1038/35101614

[62] Lam M.M., Seemann T., Bulach D.M., Gladman S.L., Chen H., Haring V., Moore R.J., Ballard S., Grayson M.L., Johnson P.D. Comparative analysis of the first complete Enterococcus faecium genome. J. Bacteriol. 2012; 194:2334–2341. 22366422 10.1128/JB.00259-12 PMC3347086

[63] Bateman B.T., Donegan N.P., Jarry T.M., Palma M., Cheung A.L. Evaluation of a tetracycline-inducible promoter in Staphylococcus aureus in vitro and in vivo and its application in demonstrating the role of sigB in microcolony formation. Infect. Immun. 2001; 69:7851–7857. 11705967 10.1128/IAI.69.12.7851-7857.2001 PMC98881

