# Supplementary material for: A novel Staphylococcus aureus cis–trans type I toxin–antitoxin module with dual effects on bacteria and host cells
Source: Nucleic Acids Res. 2018 Dec 14;47(4):1759–73. doi: 10.1093/nar/gky1257 (PMC6393315; doi:10.1093/nar/gky1257)
Supplement: Supplementary Data [file gky1257_supplemental_files.zip › supplemental_figures anf tables_Amiot_.pdf]

A

```
SprA1            1 GTCGCCTATC-TCTCAGGCGTCAATTTAGACGCAGAGAGGAGGTGTATAA     49
                   ||||||||.| ||||.|||||.|.|||| |||||||||||||||||||.|
SprA2            1 GTCGCCTAACATCTCGGGCGTTACTTTA-ACGCAGAGAGGAGGTGTATGA     49

SprA1           50 GGTGATGCTT--ATT--TTCGTTCACATCATAGCACCAGTCATCAGTGGC     95
                   .| |||| ||  |||  ||..||.|||||||..|..|||..||.||.|||
SprA2           50 CG-GATG-TTCAATTTATTAATTAACATCATGACTTCAGCTATAAGCGGC     97

SprA1           96 TGTGCCATTGCGTTTTTTTCTTATTGGCTAAGTAGACGCAA-TACAAAAT    144
                   |||....|||||||||||.|..|||||.||.|.|..||||| .|.||||.
SprA2           98 TGTCTTGTTGCGTTTTTTGCACATTGGTTACGAACGCGCAACAATAAAAA    147

SprA1          145 AGGTGACATATAGCC-GCACCAATAAAAATCCCCTCACTACCGCAAATAG    193
                   ||||||||.|||.|| .||||.|.|.||||||||||||||..|| |.|||
SprA2          148 AGGTGACAAATAACCAACACCTAAATAAATCCCCTCACTATGGC-AGTAG    196

SprA1          194 TGAGGGGA-TTGGTGT-    208
                   |||||||| |||||||
SprA2          197 TGAGGGGATTTGGTGTA    213
```

B

```
SprA1AS            1 -ACAACGAAAATAAGTATTTACTTATACACC-AATCCCCTCACTATTTGC     48
                      |||....|||| .|.|..|....||||||| ||||||||||||| .|||
SprA2AS            1 TACATAATAAAT-TGAACATCTAAATACACCAAATCCCCTCACTA-CTGC     48

SprA1AS           49 GGTAGTGAGGGG-     60
                     ..||||||||||
SprA2AS           49 CATAGTGAGGGGA     61
```

Overall nucleotide sequence identity between *sprA1*/$sprA1_{AS}$ and *sprA2*/$sprA2_{AS}$ loci = 75%

C

```
pepA1   ML-IFVHIIAPVISGCAIAFFSYWLSRRNTK---- 30
pepA2   MFNLLINIMTSAISGCLVAFFAHWLRTRNNKKGDK 35
        *: ::::*::..**** :***::**  **.*
```

50% of identity

Figure S1: Sequence alignment between *sprA2*/$sprA2_{AS}$ and *sprA1*/$sprA1_{AS}$ and alignment of PepA1 with putative PepA2 peptide. Alignments were performed using ClustalX with the needle method. (A) Alignment of *sprA1* with *sprA2*. (B) Alignment between $sprA1_{AS}$ and $sprA2_{AS}$. (C) Alignment of PepA1 with PepA2

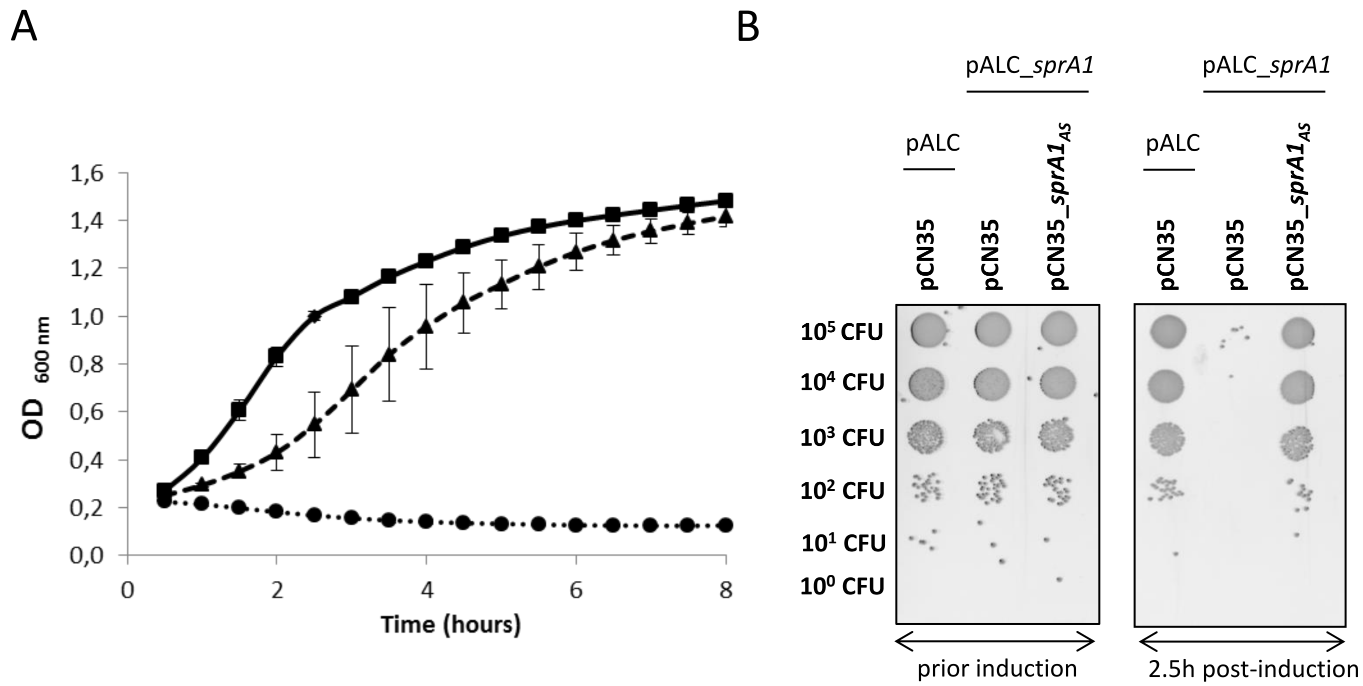

Figure S2 : *sprA1*/ *sprA1*$_{AS}$ encodes a functional toxin antitoxin system. (A) comparative growth curves, after aTc 1 µM induction, of *Staphylococcus aureus Newman* containing either empty plasmids pALC and pCN35 (squares), pALC_*sprA1*pCN35 (circles) or pALC_s*prA1*pCN35_*sprA1*$_{AS}$ (triangles). (B) Comparative viability, prior and 2.5 hours post-induction aTc 1µM, of *Staphylococcus aureus Newman* containing either empty plasmids pALCpCN35, pALC_*sprA1*pCN35 and pALC_*sprA1*pCN35*sprA1*$_{AS}$. All cultures (broth and agar) contained appropriate concentrations of chloramphenicol and erythromycin for plasmid maintenance.

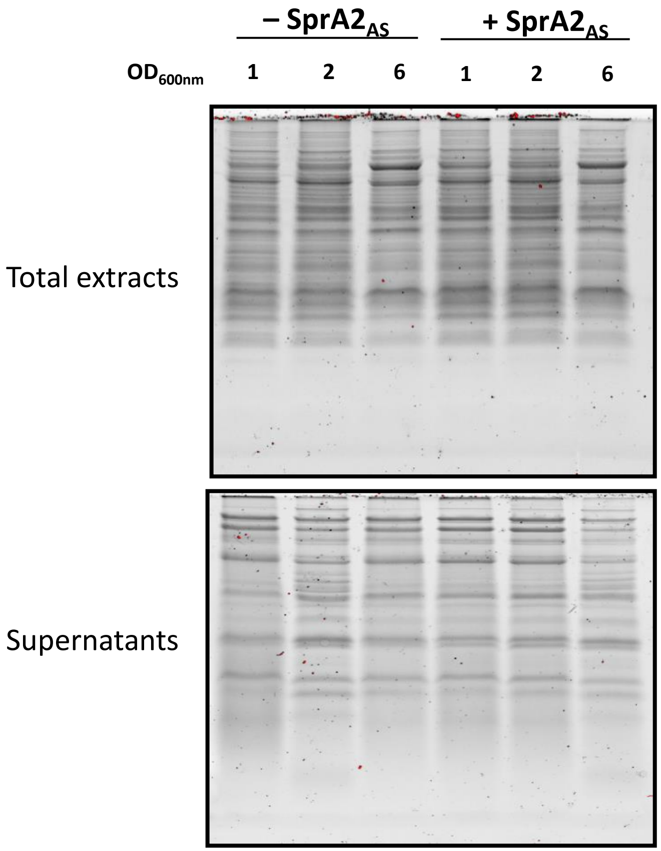

Figure S3 : Bacterial toxicity is linked to the expression of PepA2. Loading control of Western blot presented on figure 3A. Staining was performed using SYPRO Ruby.

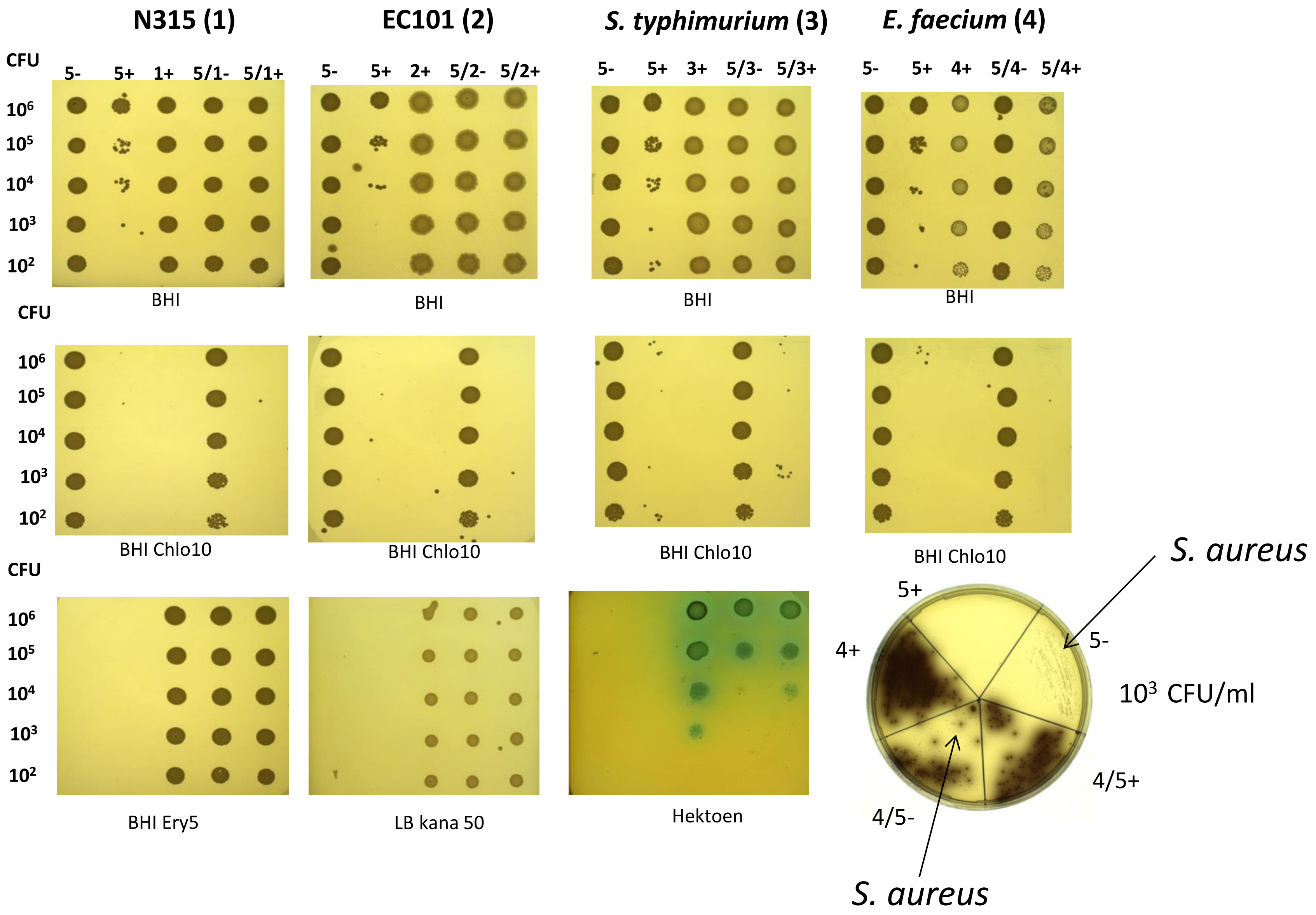

Figure S4 : Viability assay after 2.5 hours of induction with 1 µM aTc (+) or its control without (-) aTc. (1) *S. aureus* N315, (2) *E. coli* EC101, (3) *S. typhimurium*, (4) *E. faecium*, (5) *S. aureus* Newman pALC_*sprA2*.

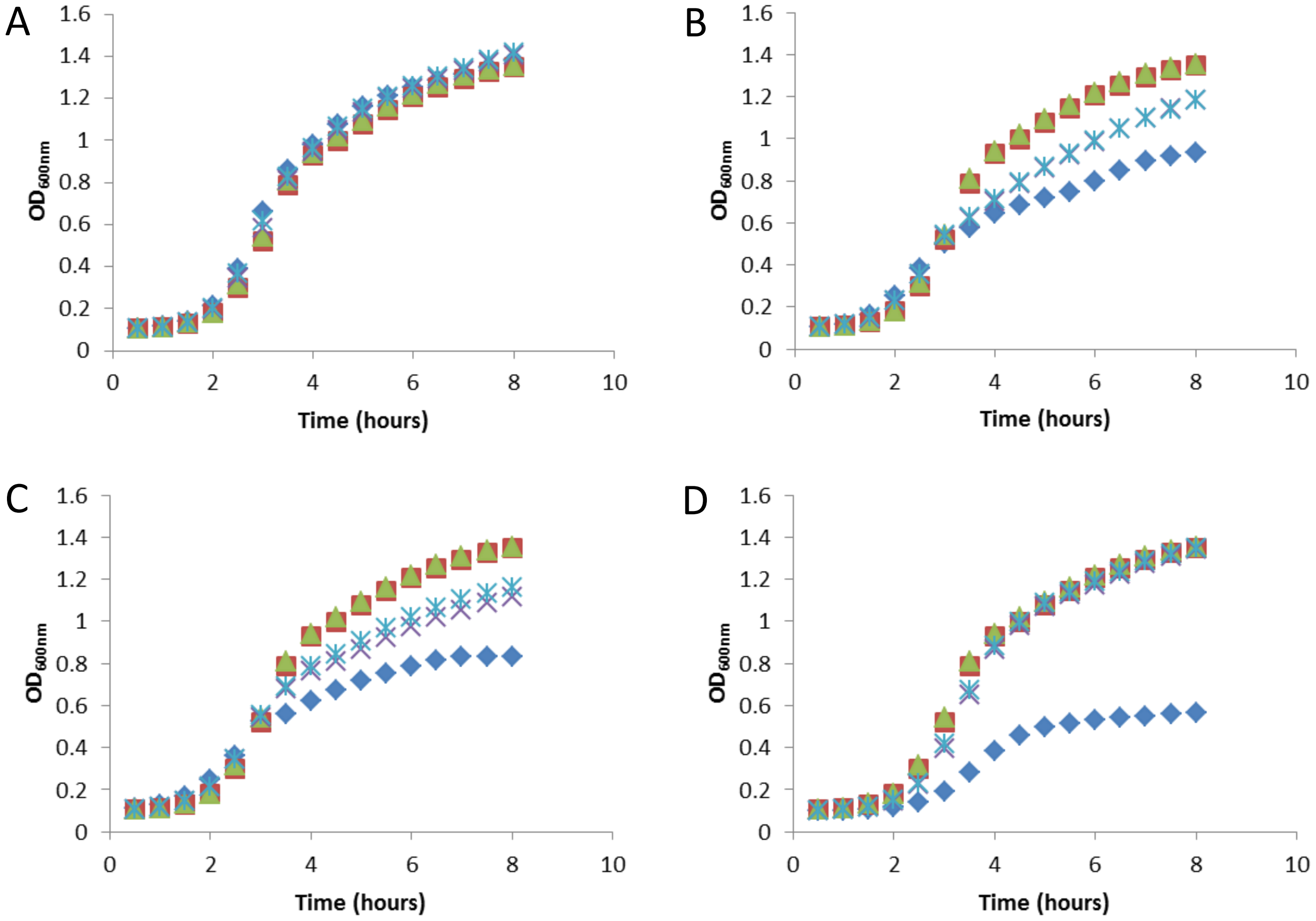

Figure S5 : SprA2 toxin does not other kill bacteria during co-cultures **(A)** Effect of SprA2 expression on *S. aureus* N315 growth: *S. aureus* N315 (blue diamonds), Newman pALC (red squares), Newman pALC_*sprA2* (green triangles), Newman pALC + N315 (purple crosses), Newman pALC_*sprA2* + N315 (blue crosses) **(B)** Effect of *sprA2* expression on *Escherichia coli* growth : *E. coli* (blue diamonds), Newman pALC (red squares), Newman pALC_*sprA2* (green triangles), Newman pALC + *E. coli* (purple crosses), Newman pALC_*sprA2* + *E. coli* (blue crosses) **(C)** Effect of *sprA2* expression on *Salmonella typhimurium* growth : *S. typhimurium* (blue diamonds), Newman pALC (red squares), Newman pALC_*sprA2* (green triangles), Newman pALC + *S. typhimurium* (purple crosses), Newman pALC_*sprA2* + *S. typhimurium* (blue crosses) **(D)** Effect of *sprA2* expression on *Enterococcus faecium* growth : *E. faecium* (blue diamonds), Newman pALC (red squares), Newman pALC_*sprA2* (green triangles), Newman pALC + *E. faecium* (purple crosses), Newman pALC_*sprA2* + *E. faecium* (blue crosses). Growth was conducted in a Biotek instrument and without aTc. The data presented are the mean of three independent experiments.

	S. aureus Newman	*S. aureus* N315	*E. coli*	*S. typhimurium*	*E. faecium*
PepA2	> 128 µM	> 128 µM	> 64 µM	> 128 µM	~ 32 µM
Vancomycin	< 0.25 µM	~ 0.25 µM	N/A	N/A	< 0.25 µM
Polymyxin B sulfate	N/A	N/A	~ 0.5 µM	~ 0.5 µM	N/A

Figure S6: Determination of MIC using synthetic PepA2 peptide. Bacteria were precultured in Mueller-Hinton broth. Overnight cultures were diluted 100 times in Mueller-Hinton broth and then cultured at 37°C until reaching an OD_{600nm} of 0.1. Cells were again diluted 100 times in Mueller-Hinton broth. 50µl of bacterial suspension were distributed into 96-well plates in the presence of 50 µl of various concentration of PepA2. Cells were cultured in a Biotek intrument for 24 hours under agitation and at 37°C.

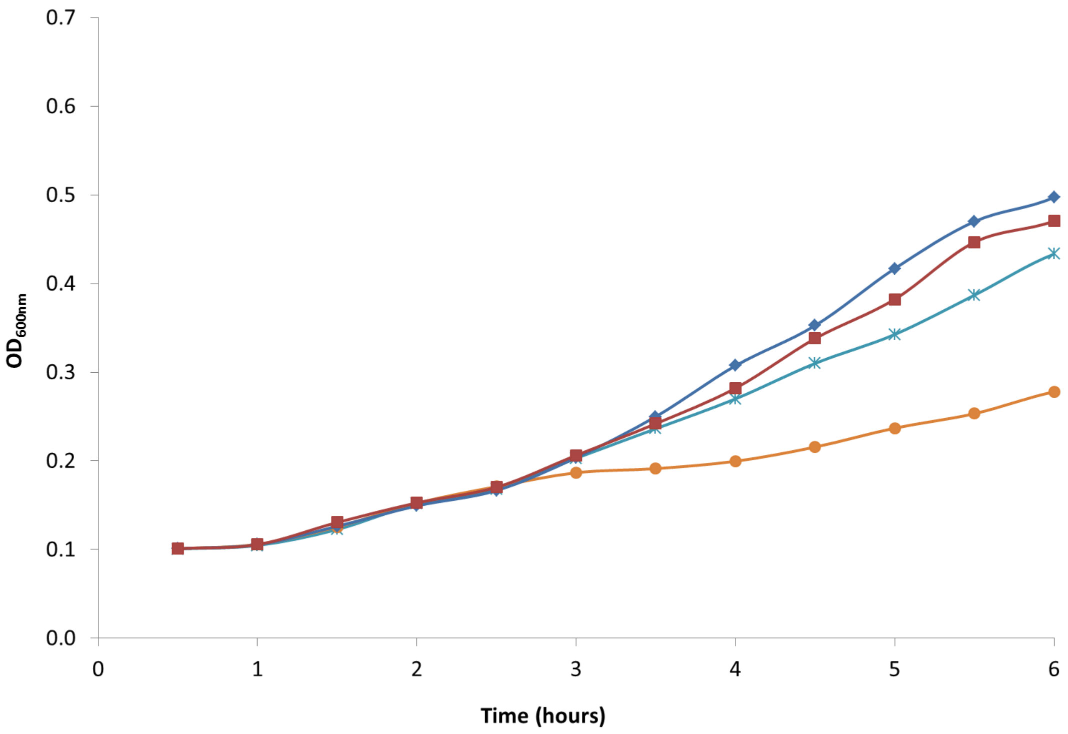

Figure S7 : SprA2 inhibits *E. coli* growth internally. Effect of SprA2 expression on *E. coli* XL1 blue growth: XL1 blue – Atc (blue diamonds), XL1 blue + Atc (red squares), XL1 blue pALC_*sprA2* – Atc (blue crosses), XL1 blue pALC_*sprA2* + Atc (orange circles). Growth was conducted in a Biotek instrument and 1 µM aTc added after 2.5 hours of growth.

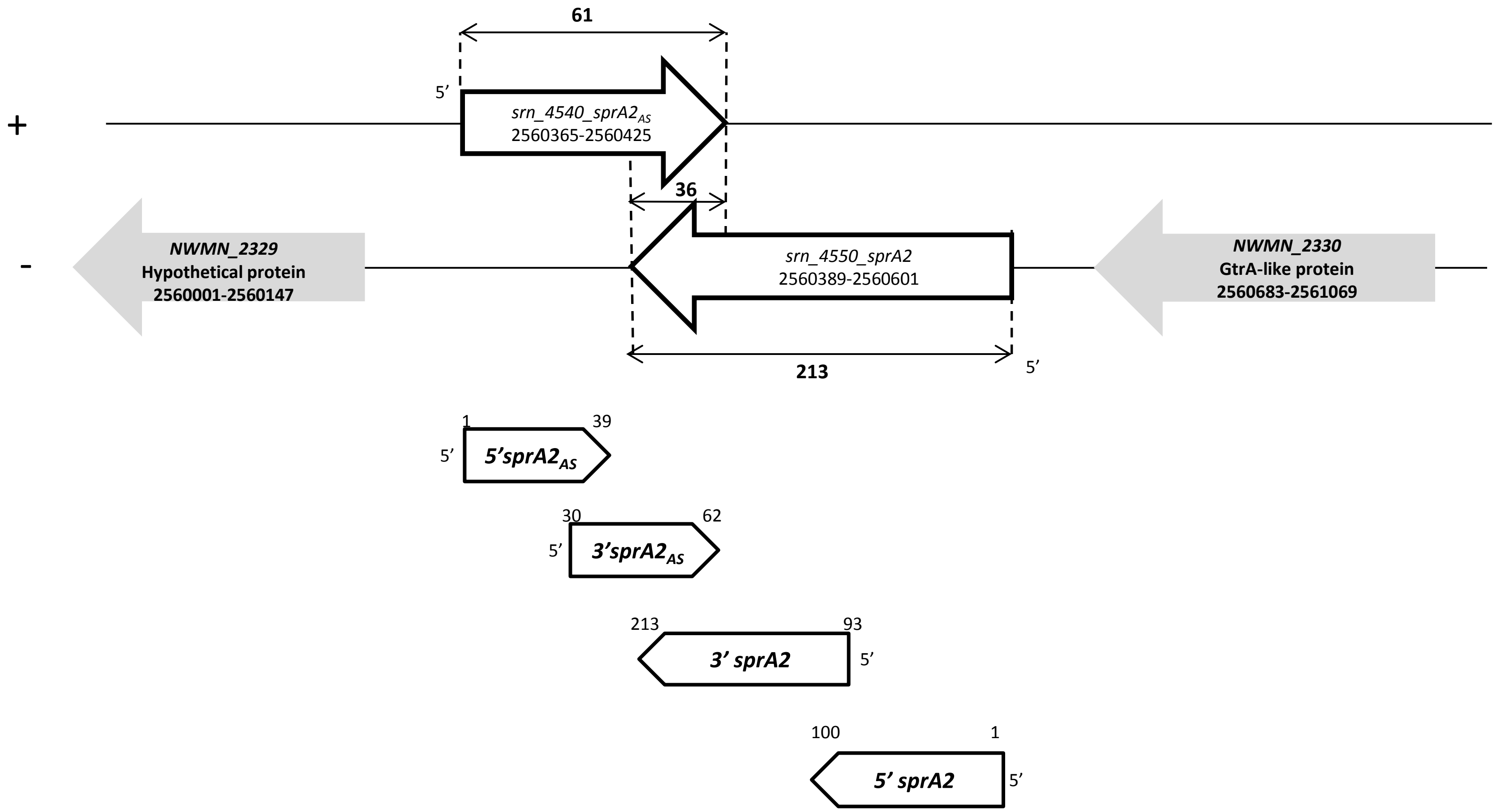

Figure S8: Synthetic RNA constructs made to demonstrate *in vitro* that the non-overlapping domains of SprA2 and $SprA2_{AS}$ interact together.

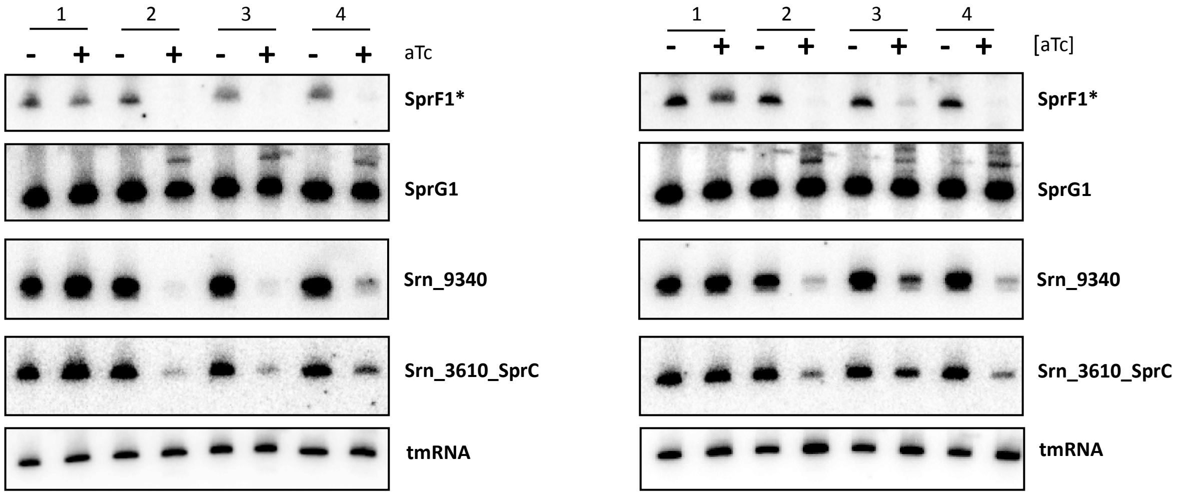

Figure S9: RNA expression profiles after 30 min of induction with 100 nM aTc. **Left panel**: 1- Newman pALC + pCN35, 2- Newman pALC_*sprA2* + pCN35, 3- Newman pALC_*sprA2* + pCN35_*sprA1*$_{AS}$, 4- Newman pALC_*sprA2* + pCN35_*sprA2*$_{AS}$. **Right panel:** 1-Newman pALC + pCN35, 2- Newman pALC_*sprA1* + pCN35, 3- Newman pALC_*sprA1* + pCN35_*sprA1*$_{AS}$, 4- Newman pALC_*sprA1* + pCN35_*sprA2*$_{AS.}$ (Half-life of Srn_9340 : 5 minutes; half-life of Srn_3610_SprC : 20 minutes).

Table S1: Primers used in this study

Name	Sequence	Target	Comment
pALCSprA2F	GTACGTGGTACCGTCGCCTAACATCTCGGGCG	sprA2	overexpression of SprA2 in pALC2073
pALCSprA2R	GTACGTGAATTCTACACCAAATCCCCTCACTAC	sprA2	overexpression of SprA2 in pALC2073
pALCSprA1F	GTACGTGGTACC GTCGCCTATCTCTCAGGCGT	sprA1	overexpression of SprA1 in pALC2073
pALCSprA1R	GTACGTGAATTCACACCAATCCCCTCACTATTT	sprA1	overexpression of SprA1 in pALC2073
98_sprA2_F_EcoRI	AAGAATTCATGGATTTTAAAAGATAGCAATT	sprA2/sprA2$_{AS}$	add FLAG epitope to SprA2
101_sprA2_R-AS_BamHI	AAGGATCCCATCTAAATACACCAAATCCC	sprA2	add FLAG epitope to SprA2
102_sprA2_R+AS_BamHI	AAGGATCCTCTCTTTGTTGGGTCCCT	sprA2/sprA2$_{AS}$	add FLAG epitope to SprA2
103_5p_Flag_R	CTTGTCATCGTCATCCTTGTAGTCGATGTCATGATCTTTATAATCACCGTCATGGTCTTTGTAGTCCATCCGTCATACACCTCC	sprA2/sprA2$_{AS}$	add FLAG epitope to SprA2
104_5p_Flag_F	GACTACAAAGACCATGACGGTGATTATAAAGATCATGACATCGACTACAAGGATGACGATGACAAGTTCAATTTATTAATTAACATCATGA C	sprA2/sprA2$_{AS}$	add FLAG epitope to SprA2
SprA2 Mut A53F1	GTACGTGGTACCGTCGCCTAACATCTCGGGCGTTACTTTAACG	sprA2	mutation in sprA2 start codon
SprA2 Mut TAGR	AATAAATTGAACTACCGTCATACACCTCCTCTCTGCGTTAAAGTAACGCCCGAGA	sprA2	mutation in sprA2 start codon
SprA2 Mut TAGF	GACGGTAGTTCAATTTATTAATTAACATCATGAC	sprA2	mutation in sprA2 start codon
SprA2 Mut G52R	AATAAATTGAACATGCGTCATACACCTCCTCTCTGCGTTAAAGTAACGCCCGAGA	sprA2	mutation upstream sprA2 start codon
SprA2 Mut G52F	GACGCATGTTCAATTTATTAATTAACATCATGAC	sprA2	mutation upstream sprA2 start codon
sens newman AS1	CGATCGGTACCAATCATTTGCGCAATCGAATATTA	SprA1$_{AS}$	express antitoxin under its own promoter in pCN35
antisens newman AS1	CGATCGGATCCTCCCCTCACTACCGCAAATAGTGA	SprA1$_{AS}$	express antitoxin under its own promoter in pCN35
sens newman AS2	CGATCGGTACCATTTCTCTTTGTTGGGTCCCTGAA	SprA2$_{AS}$	express antitoxin under its own promoter in pCN35
antisens newman AS2	CGATCGGATCCTCCCCTCACTATGGCAGTAGTGAG	SprA2$_{AS}$	express antitoxin under its own promoter in pCN35
pAS2_BamHI_5’	GGCCGGATCCTTTCTCTTTGTTGGGTCCCTG	PsprA2$_{AS}$	transcriptional fusion of PsprA2$_{AS}$ with blaZ in pCN41
pAS2_EcoRI_3’	GGCCGAATTCATTAATTATAGCATTGATGAAC	PsprA2$_{AS}$	transcriptional fusion of PsprA2$_{AS}$ with blaZ in pCN41
sensPUc18AS2	GATCGAAGCTTTAATACGACTCACTATAGGTACATAATAAATTGAACATCTAAATACACCAAATCCCC	SprA2$_{AS}$	in vitro transcription of SprA2$_{AS}$ from pUC18
antisensPUc18AS2	GATCGGAATTCCCCGGCCTCCCCTCACTATGGCAGTAGTGAGGGGATTTGGTGTATT	SprA2$_{AS}$	in vitro transcription of SprA2$_{AS}$ from pUC18
Primer Forward NEWMAN sprA2	GATCGAAGCTTTAATACGACTCACTATAGGGTCGCCTAACATCTCGGGCG	sprA2	in vitro transcription of SprA2 from pUC18
Primer Reverse NEWMAN sprA2	GATCGGAATTCCCCGGCCTACACCAAATCCCCTCACTAC	sprA2	in vitro transcription of SprA2 from pUC18
sensPUc18AS1	GGGGGGACGTCTAATACGACTCACTATAGAAAATAAGTATTTACTTATACACCAATCCCCTCACT	SprA1$_{AS}$	in vitro transcription of SprA1AS from pUC18
antisensPUc18AS1	CCCCCGAATTCCCCCGGCCCCTCACTACCGCAAATAGTGAGGGGATTGGTGTATAAGTAAATACT	SprA1$_{AS}$	in vitro transcription of SprA1AS from pUC18
sensPUc18SprA1	GGGGAATTCTAATACGACTCACTATAGTCGCCTATCTCTCAGGC	sprA1	in vitro transcription of SprA1 from pUC18
antisensPUc18SprA1	GGGAAGCTTGGGTACACCAATCCCCTCACTA	sprA1	in vitro transcription of SprA1 from pUC18
SprA2 NW R 110	GATCGGAATTCCCCGGCCACAGCCGCTTATAGCTGAAG	5' sprA2	in vitro transcription of 5' SprA2 from pUC18
SprA2 NW F 93	GATCGAAGCTTTAATACGACTCACTATAGGGCGGCTGTCTTGTTGCGTTTTTTG	3' sprA2	in vitro transcription of 3' SprA2 from pUC18
SprA2AS2 NW R 39	GATCGGAATTCCCCGGCCGGGGATTTGGTGTATTTAGA	5' SprA2$_{AS}$	in vitro transcription of 5' SprA2$_{AS}$ from pUC18
SprA2AS2 NW F 30	GATCGAAGCTTTAATACGACTCACTATAGGCCAAATCCCCTCACTACTGCC	3' SprA2$_{AS}$	in vitro transcription of 3' SprA2$_{AS}$ from pUC18
Atl 151for	TAATACGACTCACTATAGGGAATAATTAATAAATGTTAGGA	Atl 151	in vitro transcription of Atl151
Atl 151rev	TATTAGTAGTTTGATCTTGTG	Atl 151	in vitro transcription of Atl151
SprCfor	TAATACGACTCACTATAGGGAAGTCAACGACCATGCGTGGACAG	SprC	in vitro transcription of SprC
SprCrev	CCATGATTTCGAAGTCTTCATAAACTG	SprC	in vitro transcription of SprC
antisens-sprA2-Emilie	TTTAGATGTTCAATTTATTATGTAA	SprA2$_{AS}$	Northern blot probe
sprA2 total R1	TTTATTGTTGCGCGTTCGTA	SprA2	Northern blot probe
SprA1 NB 130	ATGACTGGTGCTATG	SprA1	Northern blot probe
AS1 NB 131	AGTAAATACTTATTTTCGTT	SprA1$_{AS}$	Northern blot probe
SprF1-NB	TAACTTTGGCTGGTTTCGATGGTT	SprF1	Northern blot probe
SprG1-NB	ATGCCACCATAGGCACCACCTCCTT	SprG1	Northern blot probe
antiSprC	CGGCTACTACATTCGCATGT	SprC	Northern blot probe
Srn_9340	CGACTAACTCCTGGTTATGC	Srn_9340	Northern blot probe
SprA2_F1_RACE	GGTGACAAATAACCAACACC	sprA2	RACE mapping
SprA2_F2_RACE	GGCAGTAGTGAGGGGATTTG	sprA2	RACE mapping
SprA2_R2_RACE	ACGCAACAAGACAGCCGCTT	sprA2	RACE mapping
RACE_AS_F1	GCCATAGTGAGGGGATTTA	sprA2$_{AS}$	RACE mapping
RACE_AS_R1	AGTAGTGAGGGGATTTGGT	sprA2$_{AS}$	RACE mapping
RACE_AS_R2	GGTGTATTTAGATGTTCAATTTA	sprA2$_{AS}$	RACE mapping
sprA2_F_EcoRI	GAGAGAATTCGTTCATCGTATATGTAACTCC	sprA2/sprA2$_{AS}$	deletion of sprA2/sprA2$_{AS}$
sprA2_R_BamHI	GAGAGGATCCGTAATTGCATAGTAGACATC	sprA2/sprA2$_{AS}$	deletion of sprA2/sprA2$_{AS}$
3_aphA3R+5pSprA2	GCTAGATAGGGGTCCCGAGCGCGCAATATTAAGGCTTAAG	sprA2/sprA2$_{AS}$	deletion of sprA2/sprA2$_{AS}$
[illegible]	[illegible]	[illegible]	[illegible]
